# Isotopically characterised N_2_O reference materials for use as community standards

**DOI:** 10.1002/rcm.9296

**Published:** 2022-04-28

**Authors:** Joachim Mohn, Christina Biasi, Samuel Bodé, Pascal Boeckx, Paul J. Brewer, Sarah Eggleston, Heike Geilmann, Myriam Guillevic, Jan Kaiser, Kristýna Kantnerová, Heiko Moossen, Joanna Müller, Mayuko Nakagawa, Ruth Pearce, Isabell von Rein, David Steger, Sakae Toyoda, Wolfgang Wanek, Sarah K. Wexler, Naohiro Yoshida, Longfei Yu

**Affiliations:** ^1^ Laboratory for Air Pollution/Environmental Technology Empa Dübendorf Switzerland; ^2^ Department of Environmental and Biological Sciences University of Eastern Finland Kuopio Finland; ^3^ Isotope Bioscience Laboratory – ISOFYS, Department of Green Chemistry and Technology, Faculty of Bioscience Engineering Ghent University Ghent Belgium; ^4^ National Physical Laboratory Middlesex UK; ^5^ PAGES International Project Office Bern Switzerland; ^6^ Beutenberg Campus Max‐Planck‐Institute for Biogeochemistry Jena Germany; ^7^ Air Pollution Control and Chemicals Division Federal Office for the Environment Bern Switzerland; ^8^ Centre for Ocean and Atmospheric Sciences, School of Environmental Sciences University of East Anglia Norwich UK; ^9^ Thermo Fisher Scientific Bremen Germany; ^10^ Plant Protection Chemistry Agroscope Wädenswil Switzerland; ^11^ Earth‐Life Science Institute Tokyo Institute of Technology Tokyo Japan; ^12^ Department of Chemical Science and Engineering, School of Materials and Chemical Technology Tokyo Institute of Technology Yokohama Japan; ^13^ Terrestrial Ecosystem Research, Centre for Microbiology and Environmental Systems Science University of Vienna Vienna Austria; ^14^ Institute of Environment and Ecology, Tsinghua Shenzhen International Graduate School (SIGS) Tsinghua University Shenzhen China

## Abstract

**Rationale:**

Information on the isotopic composition of nitrous oxide (N_2_O) at natural abundance supports the identification of its source and sink processes. In recent years, a number of mass spectrometric and laser spectroscopic techniques have been developed and are increasingly used by the research community. Advances in this active research area, however, critically depend on the availability of suitable N_2_O isotope Reference Materials (RMs).

**Methods:**

Within the project Metrology for Stable Isotope Reference Standards (SIRS), seven pure N_2_O isotope RMs have been developed and their ^15^N/^14^N, ^18^O/^16^O, ^17^O/^16^O ratios and ^15^N site preference (SP) have been analysed by specialised laboratories against isotope reference materials. A particular focus was on the ^15^N site‐specific isotopic composition, as this measurand is both highly diagnostic for source appointment and challenging to analyse and link to existing scales.

**Results:**

The established N_2_O isotope RMs offer a wide spread in delta (*δ*) values: *δ* ^15^N: 0 to +104‰, *δ* ^18^O: +39 to +155‰, and *δ* ^15^N^SP^: −4 to +20‰. Conversion and uncertainty propagation of *δ* ^15^N and *δ* ^18^O to the Air‐N_2_ and VSMOW scales, respectively, provides robust estimates for *δ* ^15^N(N_2_O) and *δ* ^18^O(N_2_O), with overall uncertainties of about 0.05‰ and 0.15‰, respectively. For *δ* ^15^N^SP^, an offset of >1.5‰ compared with earlier calibration approaches was detected, which should be revisited in the future.

**Conclusions:**

A set of seven N_2_O isotope RMs anchored to the international isotope‐ratio scales was developed that will promote the implementation of the recommended two‐point calibration approach. Particularly, the availability of *δ* ^17^O data for N_2_O RMs is expected to improve data quality/correction algorithms with respect to *δ* ^15^N^SP^ and *δ* ^15^N analysis by mass spectrometry. We anticipate that the N_2_O isotope RMs will enhance compatibility between laboratories and accelerate research progress in this emerging field.

## 1 INTRODUCTION

Since its first application by Sakae Toyoda and Naohiro Yoshida in 1999,^1^ site‐specific N_2_O isotope analysis has been applied by many research groups to differentiate N_2_O source and sink processes at different spatio‐temporal scales (see reviews by Toyoda et al,^2^ Ostrom et al,^3^ Decock et al,^4^ Denk et al,^5^ and Yu et al^6^). Likewise, dual‐isotope plots (e.g. *δ* ^15^N^SP^/*δ* ^15^N) or so‐called “isotope mapping” approaches have been used to constrain the contributions of specific pathways, and the effect of isotope fractionation during N_2_O reduction.^7^, ^8^ The informative value of N_2_O isotope data has been markedly increased by using the data to inform biogeochemical models, providing regional and global patterns of N_2_O losses and independent process information.^9^, ^10^, ^11^, ^12^ Advances in applications have been accompanied and accelerated by progress in analytics, complementing the traditional high‐precision isotope‐ratio mass‐spectrometry (IRMS)^1^, ^13^ by laser spectroscopic techniques, with the potential for field applicability and real‐time data coverage.^14^, ^15^, ^16^, ^17^, ^18^, ^19^

The isotopic composition of a sample is reported using the delta (*δ*) notation, which is the relative difference in isotope ratio (*R*) between a sample P and a reference material, i.e. *δ*(P/ref) = *R*_P_/*R*_ref_ − 1. For nitrogen, the ^15^N/^14^N isotope ratio is used, *R*(^15^N/^14^N) = *x*(^15^N)/*x*(^14^N), where *x* is the isotopic abundance and tropospheric N_2_ is the international reference material for the Air‐N_2_ scale. For oxygen, the ^18^O/^16^O and ^17^O/^16^O ratios are used, which are related to the Vienna Standard Mean Ocean Water (VSMOW) scale. In addition, we adopt the following notation conventions: *δ* ^15^N = *δ*(^15^N/^14^N, P/Air‐N_2_) (average of both nitrogen atoms) and *δ* ^18^O = *δ*(^18^O/^16^O, P/VSMOW). The ^15^N site preference (SP) is defined by the predominance of ^15^N substitution in the central (α) position as compared to the terminal (β) position, and calculated accordingly as *δ* ^15^N^SP^ = *δ* ^15^N^α^ − *δ* ^15^N^β^. All *δ* values in this paper are reported against Air‐N_2_ (for ^15^N/^14^N ratios) and against VSMOW (for ^18^O/^16^O and ^17^O/^16^O ratios).

Further progress in N_2_O isotope research critically depends on the compatibility of laboratory results.^20^ To achieve this, individual laboratories have to implement a traceability chain, i.e. a hierarchy of reference materials which descends with increasing uncertainty, linking the isotopic composition of primary RMs used to realise the respective scale, through secondary standards and working laboratory standards to a sample.^21^ Generally, two RMs with distinct *δ* values should be used for calibration purposes, following the two‐point data normalisation requirement. However, primary RMs and secondary scale anchors for *δ* ^15^N (ammonium sulfate, potassium nitrate) as well as *δ* ^17^O and *δ* ^18^O (water) have a different chemical identity than N_2_O sample gas. Thus, a chemical conversion reaction^20^ has to be implemented prior to analysis, which requires specialised laboratories.

The synthesis of N_2_O by thermal decomposition of isotopically characterised ammonium nitrate (NH_4_NO_3_) has been suggested as an approach to link the position‐dependent nitrogen isotopic composition of N_2_O to the Air‐N_2_ scale.^1^ The basic concept of this technique is that the nitrogen atom at the *α*‐position of of the formed N_2_O originates from NO_3_^−^, while the *β*‐nitrogen comes from NH_4_^+^.^22^ The validity of the NH_4_NO_3_ decomposition technique has been confirmed,^23^, ^24^ but its accuracy for the calibration of *δ* ^15^N^α^ and *δ* ^15^N^β^ was found to be limited by non‐quantitative NH_4_NO_3_ decomposition in combination with substantially different isotope enrichment factors of −4 or −19‰ for the conversion of the NO_3_^−^ or NH_4_^+^ nitrogen atom into the *α*‐ or *β*‐position of the N_2_O molecule.^25^ To overcome such difficulties, two new N_2_O reference gases, USGS51 and USGS52, recently became available with assigned *δ* values based on a preliminary assessment by Naohiro Yoshida and Sakae Toyoda (Tokyo Institute of Technology).^26^, ^27^ However, the two standards offer only a small range of *δ* ^15^N and *δ* ^18^O values (< 1‰), which is not suitable for a two‐point calibration approach.

In the present study, we report the development of additional N_2_O RMs within the framework of the European Metrology Programme for Innovation and Research (EMPIR) 16ENV06 project ‘Metrology for Stable Isotope Reference Standards (SIRS)’. The target isotopic composition of N_2_O RMs was selected according to discussions at a stakeholder workshop at the 19th GGMT conference at Empa (29 August 2017).^28^ The focus of this study is to extend the range of isotopic composition of N_2_O RMs compared to RMs presented in Ostrom et al^26^ and to provide additional *δ* ^17^O data in order to improve data quality/correction algorithms with respect to *δ* ^15^N^SP^ and *δ* ^15^N analysis by mass spectrometry. In addition, the link of *δ* values to the international isotope‐ratio scales was revisited.

## 2 EXPERIMENTAL

**FIGURE 1:**
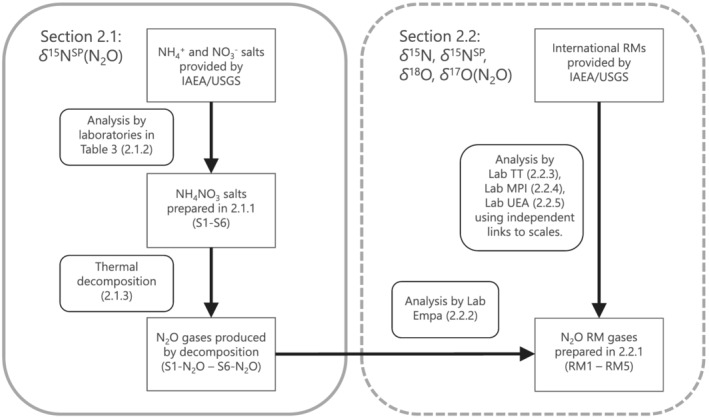
Schematic overview on the traceability chain applied in this study to propagate ^15^N/^14^N, ^18^O/^16^O and ^17^O/^16^O isotope ratios from international RMs to *δ* ^15^N, *δ* ^15^N^SP^, *δ* ^18^O and *δ* ^17^O in the novel N_2_O RMs

The main purpose of this study is the provision of isotopically characterised N_2_O RMs, covering an extended range of delta values as compared to existing gases. Figure 1 provides a schematic overview on the links established within this study between existing international RMs and the novel gaseous N_2_O RMs.

In section 2.1 (“left branch” of Figure 1), ^15^N/^14^N isotope ratios on the Air‐N2 scale were propagated from NH_4_^+^ and NO_3_^−^ salts supplied by IAEA/USGS, through isotopic analysis of gravimetrically prepared NH_4_NO_3_ salts (section 2.1.2) and their thermal decomposition (section 2.1.3), to *δ* ^15^N^β^(N_2_O) /*δ* ^15^N^α^(N_2_O) in the novel N_2_O RMs. The international RMs applied in this study are listed in Table 1. To provide a reliable link between the Air‐N2 scale and the N_2_O site‐specific isotopic composition, the NH_4_NO_3_ decomposition reaction was optimised for high yield, reproducibility, and N_2_O purity (see section 2.1.3). Following the recommended two‐point calibration approach, a number of NH_4_NO_3_ salts, ranging from ^15^N‐depleted to ^15^N‐enriched, were prepared (see section 2.1.1), decomposed, and analysed.

**TABLE 1:** International RMs applied in this study for the analysis of *δ* ^15^N(NH_4_NO_3_), *δ* ^15^N(NH_4_^+^) and *δ* ^15^N(NO_3_^−^) in NH_4_NO_3_ salts (section 2.1.2) and *δ* ^15^N as well as *δ* ^18^O in N_2_O RMs (section2.2). Values are taken from Brand et al^29^ and Ostrom et al^26^ and reported in ‰

		*δ* ^15^N_Air‐N2_	σ	*δ* ^18^O_VSMOW_	σ
**IAEA‐N‐1**	NH_4_ SO_4_	+0.43	0.07	‐	‐
**IAEA‐N‐2**	NH_4_ SO_4_	+20.41	0.12	‐	‐
**USGS25**	NH_4_ SO_4_	−30.41	0.27	‐	‐
**USGS26**	NH_4_ SO_4_	+53.75	0.24	‐	‐
**IAEA‐NO‐3**	KNO_3_	+4.72	0.13	+13.2	‐
**USGS32**	KNO_3_	+180	0	+25.4	0.2
**USGS34**	KNO_3_	−1.8	0.1	−27.78	0.37
**USGS35**	NaNO_3_	+2.7	0.1	+56.81	0.31
**USGS40**	L‐glutamic acid	−4.52	0.06	‐	‐
**USGS51**	N_2_ O	+1.21	0.21	+41.45	0.34
**USGS52**	N_2_ O	+0.29	0.25	+40.80	0.40

In section 2.2 (“right branch” of Figure 1), preparation of N_2_O RMs and analysis by expert laboratories for *δ* ^15^N(N_2_O), *δ* ^18^O(N_2_O), *δ* ^17^O(N_2_O) and *δ* ^15^N^SP^(N_2_O) is described. In one laboratory (Empa), *δ* ^15^N^SP^(N_2_O) in the N_2_O RMs was linked to the Air‐N2 scale making use of the traceability chain established in section 2.1. Links to scale applied in the other laboratories are independent and are described in detail in the respective experimental sections.

### 2.1 Re‐evaluation of NH_4_NO_3_ thermal decomposition technique to propagate *δ* ^15^N(NO_3_^−^)/*δ* ^15^N(NH_4_^+^) to *δ* ^15^N^α^(N_2_O)/*δ* ^15^N^β^(N_2_O)

#### 2.1.1 Preparation of NH_4_NO_3_ salts

Six NH_4_NO_3_ salts (S1–S6), covering a wide range of *δ* ^15^N(NH_4_^+^) and *δ* ^15^N(NO_3_^−^) values, were produced by gravimetric mixing of five commercially available NH_4_NO_3_ salts (A–E). A: unlabelled NH_4_NO_3_ (purity >98%, K299.1, Carl Roth GmbH, Karlsruhe, Germany), B: ^15^NH_4_NO_3_ (>98% ^15^NH_4_^+^, NLM‐711‐1, Cambridge Isotope Laboratories Inc., Tewksbury, USA), C: NH_4_^15^NO_3_ (>98% ^15^NO_3_^−^, NLM‐712‐1, Cambridge Isotope Laboratories Inc., Tewksbury, USA), D: ^15^NH_4_^+^‐depleted NH_4_NO_3_ (0.306% ^15^NH_4_^+^, Shoko Science Co., Ltd, Japan), E: ^15^NO_3_^−^‐depleted NH_4_NO_3_ (0.306% ^15^NO_3_^−^, Shoko Science Co., Ltd, Japan).

For preparation of these six NH_4_NO_3_ salts (Table 2), approximately 110 g of unlabelled NH_4_NO_3_ (A) was ground to a fine powder using a mortar and pestle and then dried at 120°C for 1 h (a temperature low enough to avoid triggering decomposition). From this, around 100 g (S1–S5) or around 40 g (S6) were gravimetrically (XP205, Mettler Toledo GmbH, Greifensee, Switzerland) mixed with appropriate amounts of salts B, C, D, and E to obtain the desired isotopic composition. The salt mixtures were dissolved in deionised water (Milli‐Q Advantage A10, Millipore AG, Switzerland), recrystallised, dried, and then stored in air‐tight sample containers. The isotopic homogeneity of S1–S6 was confirmed by repeated IRMS analysis (MPI‐BGC), demonstrating *δ* ^15^N(NH_4_NO_3_) values within <0.2‰ (σ, *n* = 10).

**TABLE 2:** Overview of NH_4_NO_3_ salts (S1–S6) prepared from commercially available NH_4_NO_3_ (A–E) and covering a wide range of *δ* ^15^N(NH_4_^+^) and *δ* ^15^N(NO_3_^−^) values

	Characteristic	A (unlabelled)	B (^15^NH_4_NO_3_)	C (NH_4_^15^NO_3_)	D (^15^NH_4_^+^‐depleted)	E (^15^NO_3_^−^‐depleted)
S1	Unlabelled NH_4_ NO_3_	X				
S2	^15^ NH_4_ , ^15^ NO_3_ ‐low enriched	X	X	X		
S3	Ambient isotopic composition	X	X	X		
S4	^15^ NH_4_ , ^15^ NO_3_ ‐enriched	X	X	X		
S5	^15^ NH_4_ , ^15^ NO_3_ ‐high enriched	X	X	X		
S6	^15^ NH_4_ , ^15^ NO_3_ ‐depleted	X			X	X

#### 2.1.2 Analysis of NH_4_NO_3_ salts for *δ* ^15^N(NH_4_NO_3_), *δ* ^15^N(NH_4_^+^) and *δ* ^15^N(NO_3_^−^) against IAEA and USGS RMs

Subsamples of the prepared NH_4_NO_3_ salts (S1–S6) were sent together with international reference materials ((NH_4_)_2_SO_4_, NaNO_3_, KNO_3_) provided by the IAEA (International Atomic Energy Agency, Vienna, Austria) and by USGS (U.S. Geological Survey, Reston, USA) (Table 1) to eight isotope laboratories. Table 3 provides basic information on the analytical techniques applied by the laboratories. Details on the analytics are given in the supporting information (Supplementary Method 1).

**TABLE 3:** Analytical techniques applied by the involved isotope laboratories for the analysis of *δ* ^15^N(NH_4_NO_3_), *δ* ^15^N(NH_4_^+^) and *δ* ^15^N(NO_3_^−^) in NH_4_NO_3_ salts (S1–S6). Details on the analytics are given in the supporting information (Supplementary Method 1)

Laboratory	Measurand	Technique
MPI‐BGC Lab (1)	*δ* ^15^ N(NH_4_ NO_3_ )	NH_4_ NO_3_ analysis by elemental analyser (EA)/IRMS
UC Davis Lab (2)	*δ* ^15^ N(NH_4_ NO_3_ )	NH_4_ NO_3_ analysis by EA/IRMS
University of Ghent Lab (3)	*δ* ^15^ N(NH_4_ NO_3_ ) *δ* ^15^ N(NH_4_^+^ ) *δ* ^15^ N(NO_3_^−^ )	NH_4_ NO_3_ analysis by EA/IRMS ^30^ NH_4_^+^ oxidation with BrO ^−^ to nitrite (NO_2_^−^ ), reaction with hydroxylamine (NH_2_ OH) to N_2_ O; purge‐and‐trap (PT)‐IRMS analysis ^31^ NO_3_^−^ conversion into N_2_ O by denitrifier method; PT‐IRMS analysis 32, 33
University of Pittsburgh Lab (4)	*δ* ^15^ N(NH_4_ NO_3_ ) *δ* ^15^ N(NO_3_^−^ )	NH_4_^+^ oxidation with BrO ^−^ to nitrite (NO_2_^−^ ), NO_2_^−^ + NO_3_^−^ conversion into N_2_ O by denitrifier method; PT‐IRMS analysis ^34^ NO_3_^−^ conversion into N_2_ O by denitrifier method; PT‐IRMS analysis 32, 33
UEF‐BGC Lab (5)	*δ* ^15^ N(NH_4_^+^ ) *δ* ^15^ N(NO_3_^−^ )	NH_3_ microdiffusion on acid‐impregnated glass fibre filter, followed by EA/IRMS analysis ^35^ NO_3_^−^ reaction with vanadium(III) chloride (VCl_3_ ) and sodium azide (NaN_3_ ) under acidic conditions to N_2_ O; PT‐IRMS analysis ^35^
University of Vienna Lab (6)	*δ* ^15^ N(NH_4_^+^ ) *δ* ^15^ N(NO_3_^−^ )	NH_3_ microdiffusion on acid‐impregnated glass fibre filters, followed by EA/IRMS analysis ^35^ NO_3_^−^ reaction with VCl_3_ and NaN_3_ under acidic conditions to N_2_ O; PT‐IRMS analysis ^35^
Tokyo Institute of Technology Lab (7)	*δ* ^15^ N(NH_4_^+^ ) *δ* ^15^ N(NO_3_^−^ )	NH_3_ distillation into acid solution, NH_4_^+^ oxidation with KBrO to N_2_ ; IRMS analysis ^36^ After removal of NH_4_^+^ , NO_3_^−^ reduction by Devarda's alloy to NH_4_^+^ and NH_3_ distillation; IRMS analysis as above ^36^
Hydroisotope Lab (8)	*δ* ^15^ N(NH_4_^+^ ) *δ* ^15^ N(NO_3_^−^ )	NH_4_^+^ oxidation with LiBrO to N_2_ ; IRMS analysis NH_4_^+^ removal by ion exchange; residual measured by EA/IRMS 37, 38

*δ* ^15^N(NH_4_NO_3_), *δ* ^15^N(NH_4_^+^) and *δ* ^15^N(NO_3_^**−**^) results from all laboratories were calibrated using the provided international IAEA and USGS reference materials, with *δ* ^15^N values and uncertainties according to Brand et al^29^ and references cited therein. The uncertainty of laboratory results (*σ*_cal_) was estimated from the uncertainty (*σ*_a,_ *σ*_b_) in the linear calibration function (Equation 1), considering the uncertainty in IAEA and USGS standards and their analyses, as well as the uncertainty (*σ*_meas_) in *δ* ^15^N_meas_, following the law of error propagation (Equation 2).^39^, ^40^, ^41^

(1) $$\delta^{15}N_{\mathrm{cal}}=\left(a\pm \sigma_{a}\right)\delta^{15}N_{\text{meas}}+b\pm \sigma_{b}$$

(2) $$\sigma_{\mathrm{cal}}=\sqrt{{\left(\sigma_{a}\delta^{15}N_{\text{meas}}\right)}^{2}+{\left(\sigma_{\text{meas}}a\right)}^{2}+\sigma_{b}^{2}}$$

Results (*δ* ^15^N_cal,*i*_, *σ*_cal,*i*_) from individual laboratories *i* were combined to a weighted mean value (*δ* ^15^N_weighted_, Equation 3) with an uncertainty (*σ*_weighted_, Equation 4)^42^:

(3) $$\delta^{15}N_{\text{weighted}}=\left[\frac{\delta^{15}N_{\mathrm{cal},1}}{\sigma_{\mathrm{cal},1}^{2}}+\frac{\delta^{15}N_{\mathrm{cal},2}}{\sigma_{\mathrm{cal},2}^{2}}+\ldots \right]\times {\sigma^{2}}_{\text{weighted}}\;$$

(4) $$\sigma_{\text{weighted}}=1/\sqrt{{\left(\frac{1}{\sigma_{\mathrm{cal},1}}\right)}^{2}+{\left(\frac{1}{\sigma_{\mathrm{cal},2}}\right)}^{2}+\ldots }$$

#### 2.1.3 NH_4_NO_3_ (S1–S6) thermal decomposition to N_2_O (S1‐N_2_O–S6‐N_2_O)

Aliquots of approximately 1.0 g (12.5 mmol) of NH_4_NO_3_ salts (S1–S6) were weighed into round‐bottomed glass flasks with a break‐seal (150 mL, borosilicate glass, Willi Möller AG, Zürich, Switzerland). In a variant of the NH_4_NO_3_ decomposition reaction according to Szabó et al,^43^ 1.4 g NH_4_HSO_4_ (>99.99%, Art. No. 455849‐100G, Sigma Aldrich GmbH, Buchs, Switzerland) and 0.2 g (NH_4_)_2_SO_4_ (>99.5%, Art. No. 09978‐500G, Sigma Aldrich GmbH, Buchs, Switzerland) were added. Adding surplus NH_4_^+^ salt will lead to a loss in *δ* ^15^N^β^ information but was included to test if very high reaction yields can be achieved, which might still be attractive. Therefore, for S1, both variants (with/without NH_4_HSO_4_/(NH_4_)_2_SO_4_) were tested, while for S2–S6 only decomposition without NH_4_^+^ addition was performed. Thereafter, the flasks were evacuated (<10^−1^ mbar) and flame‐sealed. The sealed flasks were placed in a circulating‐air oven (model TSW 120 ED, Salvis AG, Reussbühl Switzerland) and heated to 270°C for 24 h.^25^

After the decomposition reaction, the N_2_O product gas, e.g. S1‐derived‐N_2_O (here: S1‐N_2_O) or S6‐derived‐N_2_O (S6‐N_2_O), was purified on a vacuum manifold by cryogenic distillation. Reaction by‐ and side‐products (e.g. H_2_O, HNO_3_, NH_3_) were trapped at −78°C (dry ice/ethanol bath); N_2_O was trapped at −196°C (liquid N_2_) in a coiled stainless‐steel tube, while N_2_ and O_2_ (side products) were removed by evacuation with an oil‐sealed rotary vane pump (RV3, Edwards Ltd, Crawley, UK). Thereafter, the N_2_O product was condensed into 10 mL stainless‐steel flasks (CS‐20181323‐ARBOR, ARBOR Fluidtec AG, Wohlen, Switzerland) under liquid‐nitrogen cooling. The cryogenic extraction was repeated five times to fully capture the produced N_2_O. Finally, the N_2_O yield was determined gravimetrically (XP205 analytical balance, Mettler Toledo AG, Greifensee, Switzerland). The N_2_O purity, i.e. the absence of IR‐active impurities (<5 μmol mol^−1^ NO, <1 μmol mol^−1^ NO_2_, and <0.5 μmol mol^−1^ NH_3_), was confirmed by FTIR spectroscopy (Gasmet CX4000 FTIR gas analyser, Temet Instruments Oy, Helsinki, Finland).^44^ The distillation procedure (e.g. the trap size and the timing) was optimised for quantitative removal of N_2_ (<0.01%) and N_2_O recovery (>99.4%), using different gravimetric mixtures of high‐purity N_2_O and N_2_ (Messer Schweiz, Lenzburg, Switzerland).

##### Test for consistency of NH_4_NO_3_ decomposition reaction

First, the consistency of the NH_4_NO_3_ decomposition reaction across the large range of *δ* values (^15^N‐depleted to highly ^15^N‐enriched in S1–S6 for both salts and N_2_O) was tested. In detail, such tests were made by comparing *δ* ^15^N^α^ of NH_4_NO_3_‐derived N_2_O gases (S1‐N_2_O–S6‐N_2_O) with the *δ* ^15^N(NO_3_^−^) of substrate NH_4_NO_3_ salts (S1–S6) and *δ* ^15^N^β^ with *δ* ^15^N(NH_4_^+^), respectively. While the link provided by the NH_4_NO_3_ decomposition reaction was assumed to be valid across a wide range of *δ* values, the analytics involved in *δ* ^15^N^α^, *δ* ^15^N^β^ or *δ* ^15^N(NO_3_^−^), *δ* ^15^N(NH_4_^+^) analysis might display non‐linearities.

For this consistency test, the N_2_O gases S2‐N_2_O, S3‐N_2_O, S5‐N_2_O and S6‐N_2_O were analysed together with S1‐N_2_O and S4‐N_2_O using the QCLAS analyser (section 2.2.2). S1‐N_2_O and S4‐N_2_O were selected as calibration gases, as they differ substantially in delta values (>50‰ in *δ* ^15^N) and in preliminary experiments displayed a consistent offset between *δ* ^15^N^α^(N_2_O), *δ* ^15^N^β^(N_2_O) and *δ* ^15^N(NO_3_^−^), *δ* ^15^N(NH_4_^+^) values (data not shown). For actual *δ* ^15^N^α^ and *δ* ^15^N^β^ of S1‐N_2_O and S4‐N_2_O, known *δ* ^15^N(NO_3_^−^) and *δ* ^15^N(NH_4_^+^) values of the respective NH_4_NO_3_ salts were adopted and no correction for fractionation effects due to incomplete decomposition or branching isotope effects due to N_2_ production was applied. The uncertainty of actual *δ* ^15^N^α^ and *δ* ^15^N^β^ for S1‐N_2_O and S4‐N_2_O was estimated from the uncertainty of weighted mean *δ* ^15^N(NO_3_^−^) and *δ* ^15^N(NH_4_^+^) values (Table 5) and the standard deviation of *δ* ^15^N^α^ and *δ* ^15^N^β^ analysis for repeated decomposition experiments using the law of error propagation.

Measured *δ* ^15^N^α^ values of S1‐N_2_O and S4‐N_2_O and actual values, i.e. *δ* ^15^N(NO_3_^−^) of the educt NH_4_NO_3_ salts S1/S4, were used to define a linear calibration function (Equation 1). Then, *δ* ^15^N^α^_cal_ values were calculated from measured *δ* ^15^N^α^ values of S2‐N_2_O, S3‐N_2_O, S5‐N_2_O and S6‐N_2_O using this correction function. The combined uncertainty in *δ* ^15^N^α^_cal_ values was calculated from the uncertainty in the actual *δ* ^15^N^α^ values and the analyses of S1‐N_2_O and S4‐N_2_O, as well as the uncertainty in the measured *δ* ^15^N^α^ of the N_2_O gases S2‐N_2_O, S3‐N_2_O, S5‐N_2_O and S6‐N_2_O, in accordance with Equation 2. Finally, the agreement of *δ* ^15^N^α^_cal_ values (Equation 1) of the individual N_2_O gases (S1‐N_2_O–S6‐N_2_O) was tested against the actual *δ* ^15^N^α^ values, i.e. the *δ* ^15^N(NO_3_^−^) of the respective NH_4_NO_3_ salts (S1–S6). The same procedure was applied to *δ* ^15^N^β^ and *δ* ^15^N(NH_4_^+^).

### 2.2 Preparation of N_2_O RMs and analysis for δ^15^N(N_2_O), δ^18^O(N_2_O), δ^17^O(N_2_O) and δ^15^N^SP^(N_2_O)

#### 2.2.1 Preparation of N_2_O RMs

Currently available commercial N_2_O gases offer only limited isotopic variability. Therefore, high‐purity N_2_O (99.999%, Linde, Germany) was supplemented with defined amounts of ^15^N‐enriched/^15^N‐depleted and ^18^O‐enriched N_2_O dopant gas using a ten‐port two‐position valve (EH2C10WEPH, Valco Instruments Inc., Schenkon, Switzerland) with sample loops of different volumes (Table 4). The gas was transferred into evacuated Luxfer aluminium cylinders (3 L, 10 L, 20 L) with ROTAREX valves (Matar, Mazzano, Italy) to a final filling pressure below 45 bar to avoid condensation, given that the cylinder temperature remains above 15°C.

**TABLE 4:** Overview of N_2_O RMs produced from high‐purity N_2_O supplemented with ^**15**^N‐enriched/^**15**^N‐depleted and ^**18**^O‐enriched N_2_O

	Characteristic	High‐purity N_2_O	^15^N^14^NO	^14^N^15^NO	NN^18^O	^15^N^β^‐depl. N_2_O
RM1A/RM1B	High‐purity N_2_ O	X				
RM2	Ambient isotopic composition	X		X	X	X
RM3A/RM3B	^15^ N‐/ ^18^ O‐enriched; no SP	X	X	X	X	
RM4	^15^ N‐ / ^18^ O‐highly enriched; no SP	X	X	X	X	
RM5	^15^ N‐enriched; SP	X	X	X		

The dopant gases were commercial ^15^N^14^NO and ^14^N^15^NO (isotopic purity of >98%, Cambridge Isotope Laboratories Inc., Tewksbury, USA), as well as ^18^O‐enriched N_2_O ((36.25 ± 0.10)% NN^16^O, (63.75 ± 0.76)% NN^18^O) and ^15^N^β^‐depleted N_2_O (*δ* ^15^N^α^ = (−2.54 ± 0.005)‰, *δ* ^15^N^β^ = (−162.21 ± 0.03)‰, *δ* ^18^O = (+38.92 ± 0.003)‰), both produced and characterised at Empa. Details on the production and analysis of ^18^O‐enriched N_2_O and ^15^N^β^‐depleted N_2_O are provided in the supporting information (Supplementary Method 2). N_2_O RMs were provided to laboratories in 50 mL (Lab TT, Lab UEA) or 150 mL (Lab MPI) stainless‐steel flasks (CS‐07291113‐ARBOR, Arbor Fluidtec AG, Wohlen, Switzerland) for isotopic analysis.

#### 2.2.2 Analysis of N_2_O RMs for *δ* ^15^N^α^ and *δ* ^15^N^β^ by QCLAS at Empa (Lab Empa)

For analysis of *δ* ^15^N^α^, *δ* ^15^N^β^ and *δ* ^18^O in the N_2_O gases, a QCLAS spectrometer (Aerodyne Research Inc., Billerica, MA, USA)^45^ equipped with a continuous‐wave quantum cascade laser (cw‐QCL) with spectral emission at 2203 cm^−1^ and an astigmatic Herriott multi‐pass absorption cell (204 m path length) was applied. Prior to analysis, pure N_2_O gases (e.g. RM1–RM6, S1‐N_2_O–S6‐N_2_O) were diluted to around 50 μmol mol^−1^ using one cylinder of synthetic air ((20.5 ± 0.5)% O_2_ in N_2_, Messer Schweiz AG, Switzerland) into 2 L high‐pressure stainless‐steel cylinders (Luxfer, Messer Schweiz AG, Switzerland) using a ten‐port two‐position valve (EH2C10WEPH with a 1 mL sample loop, Valco Instruments Inc., Schenkon, Switzerland). A singular cylinder of synthetic air was used for all experiments to minimise differences in the oxygen content, which would otherwise affect pressure broadening of absorption lines, result in differences in apparent isotopologue mole fractions and increase uncertainties. The selection of synthetic air as diluent is somewhat arbitrary and not meant to represent an alternative for a full‐air matrix for high‐accuracy ambient N_2_O isotope analysis, which would enclose noble and trace gases depending on the analytics and accuracy requirements.

The spectroscopically determined isotope ratios were related to the isotope‐ratio scales realised by Toyoda et al^1^ through the analysis of calibration gases CG1 (*δ* ^15^N^α^ = (+25.73 ± 0.24)‰, *δ* ^15^N^β^ = (+25.44 ± 0.36)‰, *δ* ^18^O = (+35.86 ± 0.22)‰) and CG2 (*δ* ^15^N^α^ = (−48.59 ± 0.25)‰, *δ* ^15^N^β^ = (−46.11 ± 0.43)‰, *δ* ^18^O = (+27.37 ± 0.11)‰). The isotopic composition of the calibration gases had been previously analysed by Sakae Toyoda at the Tokyo Institute of Technology using their analytical technique as a link to the international scales.

For the analysis of N_2_O RMs by QCLAS, the site‐specific isotopic information provided by NH_4_NO_3_‐derived N_2_O gases S1‐N_2_O (*δ* ^15^N^α^ = (−1.41 ± 0.21)‰, *δ* ^15^N^β^ = (+0.33 ± 0.12)‰) and S4‐N_2_O (*δ* ^15^N^α^ = (+52.36 ± 0.15)‰, *δ* ^15^N^β^ = (+53.06 ± 0.16)‰) was propagated to the N_2_O RMs (RM1–RM5). For this, the N_2_O RMs were analysed together with S1‐N_2_O and S4‐N_2_O, as described in the preceding section, to propagate the moiety‐specific isotopic composition defined by S1 and S4 to the novel RMs (Equation 1). An uncertainty assessment was conducted according to Equation 2 including uncertainties of S1‐N_2_O and S4‐N_2_O, as discussed above, their analyses, and the analyses of RMs.

#### 2.2.3 Analysis of N_2_O RMs for *δ* ^15^N^α^, *δ* ^15^N^β^ and *δ* ^18^O by DI‐IRMS and *δ* ^17^O by HR‐IRMS at Tokyo Institute of Technology (Lab TT)

N_2_O RMs were analysed for *δ* ^15^N, *δ* ^15^N^α^, *δ* ^15^N^β^, *δ* ^18^O and *δ* ^15^N^SP^ values with a dual‐inlet (DI) MAT 252 mass spectrometer (Thermo Fisher Scientific, Bremen, Germany) against an isotopically characterised laboratory tank of pure N_2_O (N_2_O‐5N, Showa Denko, >99.999% chemical purity); C1: *δ* ^15^N = (−2.4 ± 0.4)‰, *δ* ^15^N^α^ = (−4.5 ± 0.4)‰, *δ* ^15^N^β^ = (−0.3 ± 0.8)‰, *δ* ^18^O = (+23.3 ± 1.2)‰. IRMS analysis of the N_2_O intramolecular ^15^N distribution was based on the quantification of the fragment NO^+^ (*m*/*z* 30 and 31) and molecular N_2_O^+^ (*m*/*z* 44, 45 and 46) ions to calculate isotope ratios for the entire molecule and the central (*α*) and terminal (*β*) N atom. Analysis of *δ* ^15^N (45/44) and *δ* ^15^N^α^ involves correction for interfering ^14^N_2_^17^O^+^ (*m*/*z* 45) and ^14^N^17^O^+^ (*m*/*z* 31) using actual Δ^17^O values analysed at the University of East Anglia (UEA). For the analysis of *δ* ^15^N^α^ and *δ* ^15^N^β^, rearrangement of N atoms (N^α^ and N^β^) in the ion source was considered. The *δ* ^15^N, *δ* ^15^N^α^ and *δ* ^15^N^β^ values of the local reference gas were previously anchored to Air‐N_2_ by NH_4_NO_3_ decomposition,^1^ whereas the *δ* ^18^O value was anchored to VSMOW by converting N_2_O into CO_2_ with graphite and a platinum foil (Yoshida, unpublished data). The analytical uncertainties were calculated from the uncertainty of the in‐house working N_2_O standard gases and the standard deviation for repeated measurements of the sample gas (N_2_O RM) and the in‐house working N_2_O standard following the law of error propagation. Specifically, the uncertainty of the in‐house working N_2_O standard gas for *δ* ^15^N, *δ* ^15^N^α^ and *δ* ^15^N^β^ values comprises both the uncertainty in the *δ* ^15^N(NH_4_^+^) and *δ* ^15^N(NO_3_^−^) analysis and the repeatability of the NH_4_NO_3_ decomposition reaction. For *δ* ^18^O, the uncertainty of the in‐house working N_2_O standard gas includes the repeatability of the conversion reaction of N_2_O into CO_2_ with graphite. *δ* ^17^O signatures of three N_2_O RMs (RM1A, RM3A, RM4) were analysed by high‐resolution IRMS (MAT 253 Ultra, Thermo Scientific, Bremen, Germany). Experimental details of this prototype analyses are provided in the supporting information (Supplementary Method 3).

#### 2.2.4 Analysis of N_2_O RMs for *δ* ^15^N and *δ* ^18^O by EA/IRMS and DI‐IRMS at MPI‐BGC (Lab MPI)

##### Analysis for *δ* ^15^N by EA/IRMS (MPI‐I)

*δ* ^15^N values of the N_2_O RMs were determined using a modified EA/IRMS system (EA 1110 CHN combustion analyzer, CE Instruments Ltd, Wigan, UK; Delta plus isotope ratio mass spectrometer, Thermo Fisher Scientific, Bremen, Germany). The system and the method used have been described by Sperlich et al.^46^ The *δ* ^15^N values of the sample N_2_O were scaled to IAEA‐N‐1 and USGS32. In addition to the sample gases, an in‐house standard N_2_O gas NINO was analysed in each sample run, which was used as an anchor for *δ* ^15^N measurements by DI‐IRMS. USGS40, and the in‐house standards Ali‐j3 (*δ* ^15^N = (−1.51 ± 0.1)‰; acetic anilide) and Caf‐j3 (*δ* ^15^N = (−15.46 ± 0.1)‰; caffeine), were analysed in each daily run as quality controls, but not used for data correction.

##### Analysis for *δ* ^15^N and *δ* ^18^O by dual‐inlet IRMS (MPI‐II)

The N_2_O RMs were analysed twice (September 2019, February 2021) on a DI‐IRMS system (MAT253, Thermo Fisher Scientific, Bremen, Germany) using separately subsampled flasks. We note that the published *δ* values for USGS51 and USGS52 are average values with a rather large deviation between laboratories. Therefore, we scaled the DI‐IRMS *δ* ^15^N analyses to the in‐house standard NINO using the value reported for the primary calibration using EA/IRMS (*δ* ^15^N = (+0.54 ± 0.21)‰). The *δ* ^18^O value of NINO (*δ* ^18^O = (+39.94 ± 0.34)‰) was determined by setting the *δ* ^18^O of USGS51 equal to the average value from the interlaboratory comparison (*δ* ^18^O = (+41.45 ± 0.34)‰) published by Ostrom et al.^26^ In addition, USGS52 was analysed to test the consistency of the results (shown in Table 9) but not used for correction.

In contrast to the EA/IRMS technique, where *δ* ^15^N is measured from N_2_ gas, the DI‐IRMS method allows the analyses of *δ* ^15^N and *δ* ^18^O values by simultaneously recording *m*/*z* 44 (^14^N^14^N^16^O^+^), 45 (^15^N^14^N^16^O^+^, ^14^N^15^N^16^O^+^, ^14^N^14^N^17^O^+^) and 46 (^14^N^14^N^18^O^+^, ^15^N^15^N^16^O^+^, ^14^N^15^N^17^O^+^) ion currents. *δ* ^15^N and *δ* ^18^O values for N_2_O RMs were calculated according to Kaiser et al^47^ to correct for isobaric interferences, for which the Δ^17^O values determined by UEA were used.

The uncertainty of the analyses was calculated from the uncertainty of *δ* ^15^N and *δ* ^18^O measurements of the N_2_O standard gases (NINO, USGS51) and from the standard deviation for repeated measurements of the sample gas (N_2_O RM) and the N_2_O standards, following the law of error propagation.

#### 2.2.5 Analysis of N_2_O RMs for *δ* ^15^N, *δ* ^18^O and *δ* ^17^O by IRMS at UEA (Lab UEA)

##### Analysis for *δ* ^15^N, *δ* ^18^O and *δ* ^17^O by GC/IRMS (UEA‐I)

The N_2_O RM samples and a N_2_O‐MG‐6.0 working reference (99.9999% chemical purity, N_2_O‐MG‐6.0, Messer‐Griesheim, Krefeld, Germany) were diluted to 0.09 mmol mol^−1^ in N_2_ (zero grade, BOC, UK), filled into 20 mL serum vials (Wheaton, Fisher Scientific, Loughborough, UK) and analysed for ^45^ *δ*(Ν_2_Ο) and ^46^ *δ*(Ν_2_Ο) on a custom‐built automated cryogenic extraction and purification system comprised of an autosampler, a valve system, and PoraPLOT Q pre‐ and main columns (Agilent Technologies, Santa Clara, USA), coupled to a GEO 20‐20 isotope ratio mass spectrometer (Sercon Ltd, Crewe, UK).

Using the same mass spectrometer, these samples were also analysed for ^33^ *δ*(O_2_) = *δ* ^17^O, ^34^ *δ*(O_2_) ≈ *δ* ^18^O (the error of this approximation is <0.01‰) and ^29^ *δ*(Ν_2_) = *δ* ^15^Ν after cryogenic N_2_O extraction and decomposition to N_2_ and O_2_ with a 500 mm long pure gold tube (1.6 mm OD, 0.6 mm ID; Heimerle & Meule, Pforzheim, Germany) held at 854°C. N_2_ and O_2_ were separated directly (without further cryofocussing) on a molecular‐sieve 5‐Å PLOT main column (Restek, Bellefonte, USA, 30 m × 0.32 mm, 30 μm, 30°C, 1.3 mL min^−1^ (at 20°C and 1 bar, standard temperature and pressure)). The quantitative conversion of N_2_O was verified by swapping the molecular‐sieve main column for the PoraPLOT Q main column and testing for residual N_2_O with the mass spectrometer. The raw *δ* ^17^O and *δ* ^18^O measurements were affected by scale compression. To correct for this, a logarithmic scale normalisation^48^, ^49^ was applied using the *δ* ^18^O value of +112.4‰ (relative to N_2_O‐MG‐6.0) derived from the ^46^ *δ*(Ν_2_Ο) measurements of the diluted RM4 sample measured on the GEO 20‐20 mass spectrometer. The same normalisation was used for *δ* ^17^O as for *δ* ^18^O because no N_2_O reference material with a calibrated *δ* ^17^O value was available. No scale‐normalisation was applied to the *δ* ^15^N measurements. Uncertainties were calculated using the law of error propagation from the standard deviations of replicate measurements against the working reference gas and the calibration uncertainties of the working reference gas against Air‐N_2_ and VSMOW.^42^

##### Analysis for *δ* ^15^N, *δ* ^18^O and *δ* ^17^O by dual‐inlet IRMS (UEA‐II)

The N_2_O RM samples were analysed for ^45^ *δ*(Ν_2_Ο) and ^46^ *δ*(Ν_2_Ο) with respect to the N_2_O working reference N_2_O‐MG‐6.0 using the dual‐inlet system of a Finnigan MAT 253 isotope ratio mass spectrometer (Thermo Fisher Scientific, Bremen, Germany). The N_2_O‐MG‐6.0 working reference has been calibrated by Kaiser et al,^50^ who reported values of *δ* ^15^N = (+1.01 ± 0.06)‰ with respect to Air‐N_2_, as well as *δ* ^18^O = (+38.45 ± 0.22)‰ and *δ* ^17^O = (+19.66 ± 0.11)‰ with respect to VSMOW.^51^ Actual *δ* ^17^O values of N_2_O RMs analysed with the Sercon GEO 20‐20 were used for the data correction according to Kaiser et al.^47^ Uncertainties were calculated using the law of error propagation from the standard deviations of replicate measurements against the working reference gas and the calibration uncertainties of the working reference gas against Air‐N_2_ and VSMOW.^30^

## 3 RESULTS AND DISCUSSION

### 3.1 Re‐evaluation of the NH_4_NO_3_ thermal decomposition technique to propagate *δ* ^15^N(NO_3_^−^)/*δ* ^15^N(NH_4_^+^) to *δ* ^15^N^α^(N_2_O)/*δ* ^15^N^β^(N_2_O)

In the following sections, the main procedures for anchoring of *δ* ^15^N^α^ and *δ* ^15^N^β^ in N_2_O to the Air‐N_2_ scale and calculating uncertainties are described. Section 3.1.1 details results of *δ* ^15^N(NH_4_^+^) and *δ* ^15^N(NO_3_^−^) analyses in NH_4_NO_3_ salts (S1–S6) by eight isotope laboratories against international IAEA and USGS standards. Section 3.1.2 informs about optimal conditions for NH_4_NO_3_ decomposition at high yield, repeatability, and N_2_O purity. To enable the two‐point calibration, a number of NH_4_NO_3_ salts with different isotopic composition were produced and decomposed and the consistency of *δ* ^15^N^α^ and *δ* ^15^N^β^ of the N_2_O gases (S1‐N_2_O–S6‐N_2_O) and the *δ* ^15^N(NH_4_^+^) and *δ* ^15^N(NO_3_^−^) of NH_4_NO_3_ salts (S1–S6) was tested (section 3.1.3).

#### 3.1.1 Isotopic composition of NH_4_NO_3_ salts for *δ* ^15^N(NH_4_NO_3_), *δ* ^15^N(NO_3_^−^) and *δ* ^15^N(NH_4_^+^)

The isotopic composition of the prepared NH_4_NO_3_ salts (S1–S6), as analysed by the eight isotope laboratories and calibrated to Air‐N_2_ by analysis of IAEA and USGS standards, is indicated in Table 5. The uncertainty (σ) of individual laboratory results was estimated using the law of error propagation including the uncertainty in the international standards, their analyses, and the analyses of the NH_4_NO_3_ samples (Equations 1 and 2).

**TABLE 5:** *δ* ^15^N(NH_4_NO_3_) (top), *δ* ^15^N(NO_3_^−^) (middle), and *δ* ^15^N(NH_4_^+^) (bottom) of prepared NH_4_NO_3_ salts (S1–S6) analysed by different laboratories using techniques described in Table 3 and the supporting information (Supplementary Method 1). Results from individual laboratories were calibrated using international (IAEA, USGS) standards^29^ and their uncertainties (σ) calculated following the law of error propagation. Laboratories: (1) MPI‐BGC, (2) UC Davis, (3) University of Ghent, (4) University of Pittsburgh, (5) UEF‐BGC, (6) University of Vienna, (7) Tokyo Tech, (8) Hydroisotop

*δ* ^15^N(NH_4_NO_3_)/‰	Lab (1)	Lab (2)	Lab (3)	Lab (4)^a^			σ (1)	σ (2)	σ (3)	σ (4)^a^			Weighted mean ± σ
S1	−0.60	−0.70	+0.05	+0.64			0.11	0.07	0.09	0.08			**−0.44 ± 0.05**
S2	+13.77	+13.73	+14.48	+15.11			0.17	0.15	0.10	0.07			**+14.14 ± 0.08**
S3	+7.23	+7.02	+8.11	+8.22			0.13	0.07	0.11	0.06			**+7.31 ± 0.06**
S4	+52.65	+52.42	+53.27	+54.55			0.17	0.41	0.24	0.11			**+52.81 ± 0.13**
S5	+107.56	+107.61	+108.21	+110.58			0.24	0.32	0.19	0.18			**+107.90 ± 0.13**
S6	−49.91	−50.00	−49.37	−49.25			0.13	0.14	0.24	0.13			**−49.87 ± 0.09**

*δ* ^15^ N(NO_3_^−^)/‰	Lab (3)	Lab (4)	Lab (5)	Lab (6)	Lab (7)	Lab (8)	σ (3)	σ (4)	σ (5)	σ (6)	σ (7)	σ (8)	Weighted mean ± σ
S1	−2.07	−0.78	−1.76	−1.27	−1.36	−0.09	0.23	0.66	0.70	0.13	0.14	1.03	**−1.41 ± 0.09**
S2	+12.95	+13.80	+13.57	+13.75	+13.25	+16.13	0.22	0.09	0.69	0.14	0.37	1.04	**+13.69 ± 0.07**
S3	+13.41	+14.23	+14.04	+14.15	+12.48	+15.73	0.24	0.13	0.69	0.14	0.51	1.04	**+14.04 ± 0.09**
S4	+52.03	+53.11	+53.03	+51.60	+51.79	+54.69	0.51	0.10	0.83	0.26	0.09	1.08	**+52.36 ± 0.07**
S5	+112.78	+114.33	+114.86	+114.42	+112.02	+117.50	1.67	0.16	1.42	0.39	2.30	1.24	**+114.37 ± 0.15**
S6	−51.44	−50.54	−51.07	−50.64	−50.37	−48.48	0.46	0.14	0.94	0.34	0.11	1.07	**−50.47 ± 0.08**

*δ* ^15^ N(NH_4_^+^ )/‰	Lab (3)	Lab (5)	Lab (6)	Lab (7)	Lab (8)		σ (3)	σ (5)	σ (6)	σ (7)	σ (8)		Weighted mean ± σ
S1	+0.21	+1.02	+0.15	+0.96	+1.12		0.59	0.35	0.10	0.23	0.70		**+0.33 ± 0.09**
S2	+13.59	+14.72	+13.97	+14.99	+15.65		1.18	0.58	0.15	0.28	0.72		**+14.26 ± 0.13**
S3	+0.19	+1.31	+0.14	+0.74	+0.71		0.77	0.45	0.08	0.97	0.70		**+0.19 ± 0.08**
S4	+52.85	+52.17	+52.32	+53.14	+53.34		1.01	1.36	0.27	0.09	0.91		**+53.06 ± 0.08**
S5	+99.18	+100.35	+101.43	+102.82	+100.08		1.40	1.08	0.41	1.16	1.29		**+101.22 ± 0.34**
S6	−49.34	−48.84	−49.13	−47.35	−47.34		0.98	0.74	0.21	1.70	0.86		**−49.01 ± 0.19**

^a^ Results were not considered for calculation of weighted mean values as the applied technique is associated with a higher uncertainty.

For *δ* ^15^N(NH_4_NO_3_), all results obtained by EA/IRMS were included for calculation of the weighted mean value except for results by one laboratory (Lab 4), as this laboratory used a more complicated analytical procedure with higher uncertainties. For *δ* ^15^N(NO_3_^−^) and *δ* ^15^N(NH_4_^+^), all laboratory results were included to calculate the weighted mean values, irrespective of the applied analytical technique.

A comparison of *δ* ^15^N(NH_4_NO_3_) with average *δ* ^15^N(NH_4_^+^)/*δ* ^15^N(NO_3_^−^) values indicates a good agreement to within <0.2‰. Nonetheless, results by individual laboratories for moiety‐specific *δ* values deviate substantially from the weighted mean. As an example, *δ* ^15^N(NO_3_^−^) results from Lab 8 are substantially higher than those from other laboratories by an average of (+2.15 ± 0.58)‰. This may be due to the specific preparation technique applied, NH_4_^+^ removal by ion exchange, a technique which is prone to preferential retention/elution of ^15^NO_3_.^52^ In contrast, microdiffusion methods tend to underestimate *δ* ^15^N values of both NO_3_^−^ and NH_4_^+^, which may be reflected in the *δ* ^15^N(NH_4_^+^) values of Lab 6 but not those of Lab 5, where a similar technique was used. Conversely, systematic fractionation effects by preparation techniques should be accounted for by identical treatment (IT) of the provided IAEA and USGS standards used for data correction. In summary, analysis of *δ* ^15^N(NH_4_^+^) and *δ* ^15^N(NO_3_^−^) is still challenging; however, the ensemble of techniques applied in this study provides good agreement with *δ* ^15^N(NH_4_NO_3_) values.

#### 3.1.2 Optimal reaction conditions for NH_4_NO_3_ thermal decomposition to N_2_O

Under optimised reaction conditions (270°C, 24 h) and distillation procedure, an average N_2_O yield of 93–95% was achieved for the decomposition of NH_4_NO_3_ salts S1–S6 (Table S1, supporting information). The yield and repeatability of the decomposition reaction are somewhat better than reported in our earlier study (91.2–93.5%)^25^ and published by Toyoda et al^1^ ((90.1 ± 3.7)%, *n* = 3), surpasses results by Westley et al^23^ ((65.6 ± 5.1)%, *n* = 20). A further increase in the yield of the NH_4_NO_3_ decomposition was achieved by conducting the reaction in a NH_4_HSO_4_–(NH_4_)_2_SO_4_ melt (around 2%), as suggested for industrial applications by Szabó et al.^43^ This variant displayed comparable *δ* ^15^N^α^ values but a loss in the *δ* ^15^N^β^ information due to NH_4_^+^ salt addition, and was thus not continued.

No correction was applied to *δ* ^15^N^α^ and *δ* ^15^N^β^ for the loss in N_2_O (around 5–7%), mainly due to uncertainties in the reaction mechanisms (incomplete decomposition or side‐reaction), which makes it difficult to estimate the effect on *δ* values. Assuming incomplete reaction accompanied by fractionation effects, according to our earlier study,^25^ a 5% reduction in yield for S1–S6 should result in 0.7/3.0/1.8‰ lower *δ* ^15^N^α^/*δ* ^15^N^β^/*δ* ^15^N values, respectively. However, a much smaller difference in *δ* ^15^N was observed when comparing results of N_2_O RMs analysed by QCLAS (calibrated by NH_4_NO_3_ decomposition) with IRMS analyses. Therefore, our assumption is that the decrease in yield is at least partly caused by a “branching” side reaction, e.g. nitrogen gas (N_2_) production,^53^ which was observed to display higher *δ* ^15^N(N_2_) values.^1^ We speculate that N_2_ production has a minor effect on *δ* ^15^N^α^, *δ* ^15^N^β^ and *δ* ^15^N^SP^, but the effect is expected to depend on the timing of N_2_ generation, which is not known.

#### 3.1.3 Consistency of isotopic composition of S1‐N_2_O–S6‐N_2_O

A general goal of the current project was to provide a link to the Air‐N_2_ scale and to determine the N_2_O site‐specific isotopic composition across a wide range of *δ* values. Therefore, the consistency of the isotopic composition of the N_2_O gases (*δ* ^15^N^β^ and *δ* ^15^N^α^, S1‐N_2_O–S6‐N_2_O) and the NH_4_NO_3_ salts (*δ* ^15^N(NH_4_^+^) and *δ* ^15^N(NO_3_^−^), S1–S6) was tested. The detailed procedure is described in section 2.1.3. In short, assuming the validity of the NH_4_NO_3_ decomposition reaction, measured *δ* ^15^N^α^ values of S1‐N_2_O/S4‐N_2_O and actual *δ* ^15^N^α^ values, i.e. *δ* ^15^N(NO_3_^−^) of the educt NH_4_NO_3_ salts S1/S4, were used to define a linear calibration function. *δ* ^15^N^α^_cal_ values of S2‐N_2_O, S3‐N_2_O, S5‐N_2_O and S6‐N_2_O were calculated from measured *δ* ^15^N^α^ using this correction function and compared against actual values (Table 6).

**TABLE 6:** Consistency check for *δ* ^15^N^α^_cal_, *δ* ^15^N^β^_cal_, *δ* ^15^N^SP^_cal_ and *δ* ^15^N_cal_ of N_2_O gases (S2‐N_2_O, S3‐N_2_O, S5‐N_2_O, S6‐N_2_O) as analysed by QCLAS and referenced to the actual isotopic composition of S1‐N_2_O and S4‐N_2_O; against the actual isotopic composition of the same gases, expressed by *δ* ^15^N(NO_3_^−^), *δ* ^15^(NH_4_^+^), *δ* ^15^N(NO_3_^−^)–*δ* ^15^N(NH_4_^+^) and *δ* ^15^N(NH_4_NO_3_) of the respective NH_4_NO_3_ substrates (S2, S3, S5, S6). For details see section 2.1.3. The number of repetitions (*n*) for S2‐N_2_O/S3‐N_2_O analysis is 3, for S5‐N_2_O and S6‐N_2_O it is 10. All values are reported in ‰

	Isotopic composition of N_2_O as analysed by QCLAS (Sx‐N_2_O)							
	*δ* ^15^N^α^_cal_	σ	*δ* ^15^N^β^_cal_	σ	*δ* ^15^N^SP^_cal_	σ	*δ* ^15^N_cal_	σ
S2‐N_2_ O	+13.20	0.23	+13.99	0.29	−0.79	0.37	+13.60	0.37
S3‐N_2_ O	+13.70	0.17	+0.36	0.24	+13.34	0.30	+7.03	0.30
S5‐N_2_ O	+113.53	0.24	+103.67	0.32	+9.78	0.41	+108.60	0.41
S6‐N_2_ O	−51.26	0.17	−50.03	0.24	−1.27	0.30	−50.60	0.30

	Actual isotopic composition derived from NH_4_ NO_3_ (Sx)							
	*δ* ^15^ N(NO_3_^−^ )	σ	*δ* ^15^ N(NH_4_^+^ )	σ	*δ* ^15^ N(NO_3_^−^ ) *− δ* ^15^ N(NH_4_^+^ )	σ	*δ* ^15^ N(NH_4_ NO_3_ )	σ
S2	+13.69	0.07	+14.26	0.13	−0.57	0.15	+14.14	0.08
S3	+14.04	0.09	+0.19	0.08	+13.85	0.12	+7.31	0.06
S5	+114.37	0.15	+101.22	0.34	+13.16	0.37	+107.90	0.13
S6	−50.47	0.08	−49.01	0.19	−1.46	0.21	−49.87	0.09

Results of *δ* ^15^N^α^_cal_/*δ* ^15^N^β^_cal_/*δ* ^15^N^SP^_cal_ for S2‐N_2_O and S3‐N_2_O agree within expanded uncertainties (2 x σ_cal_, Equation 2) with the isotopic composition of the substrate NH_4_NO_3_ (S2, S3; Table 5). In contrast, for S5‐N_2_O and S6‐N_2_O, *δ* ^15^N^α^_cal_/*δ* ^15^N^β^_cal_/*δ* ^15^N^SP^_cal_ values of the N_2_O gases show a significant deviation from *δ* ^15^N(NO_3_^−^)/*δ* ^15^N(NH_4_^+^)/*δ* ^15^N(NO_3_^−^) – *δ* ^15^N(NH_4_^+^) of the respective salts (S5, S6). Assuming similar fractionation effects for decomposition of all NH_4_NO_3_ salts (S1–S6), provided the comparable decomposition yield (Table S1, supporting information), we conclude that the deviation is caused by non‐linearities either in N_2_O isotope analysis by QCLAS or in *δ* ^15^N(NO_3_^−^) and *δ* ^15^N(NH_4_^+^) analyses of the NH_4_NO_3_ salts. The latter is more plausible, as the QCLAS analyses using the same calibration approach showed good agreement with independent IRMS measurements for N_2_O RM with high ^15^N enrichment (see RM4, Table S2, supporting information). The observed deviations were highest for *δ* ^15^N^β^_cal_ to *δ* ^15^N(NH_4_^+^) (e.g. S5), which agrees with earlier studies indicating challenges in *δ* ^15^N(NH_4_^+^) analysis, but this may also be due to the lack of available international standards for *δ* ^15^N(NH_4_^+^) that cover *δ* values above (+53.75 ± 0.24)‰ (USGS26) and below (**−**30.41 ± 0.27)‰ (USGS25).

In summary, our results demonstrate consistency of the isotopic composition of the N_2_O gases from around zero (S1‐N_2_O) to ^15^N‐enriched (S4‐N_2_O) and of the substrate NH_4_NO_3_ salts (S1–S4). Thereby, our study covers a much larger range of *δ* values (> 50‰ in *δ* ^15^N^α^_cal_ and *δ* ^15^N^β^_cal_) than earlier studies,^1^, ^23^ and provides a robust link to the Air‐N_2_ scale. At very high and low ^15^N enrichment (S5‐N_2_O, S6‐N_2_O), the calibration approach using NH_4_NO_3_ decomposition is more challenging, probably due to less satisfying analytical accuracy of *δ* ^15^N(NH_4_^+^) measurements to date. As the N_2_O gases S5‐N_2_O and S6‐N_2_O were not included in the analysis of N_2_O RMs, their enhanced uncertainty in *δ* ^15^N^α^_cal_ and *δ* ^15^N^β^_cal_ does not affect the data quality of N_2_O RMs.

### 3.2 Isotopic composition of N_2_O RMs

#### 3.2.1 Isotopic composition of N_2_O RMs for *δ* ^15^N^SP^ by QCLAS and IRMS

The novel N_2_O RMs (RM1–RM5) were calibrated against Air‐N_2_ by both QCLAS (Lab Empa) and IRMS (Lab TT) analyses. For QCLAS analyses, two N_2_O gases produced by NH_4_NO_3_ decomposition (S1‐N_2_O, S4‐N_2_O) were applied to define a calibration function and propagate the isotopic information of the NH_4_NO_3_ salts (*δ* ^15^N(NO_3_^−^), *δ* ^15^N(NH_4_^+^)) to the N_2_O RMs (*δ* ^15^N^α^, *δ* ^15^N^β^). *δ* ^15^N^SP^ and *δ* ^15^N were calculated using definitions and their uncertainty estimated using the law of error propagation. In Table 7, *δ* ^15^N^SP^ values acquired by QCLAS (Lab Empa) using the calibration approach established in this study are compared with results provided by DI‐IRMS (Lab TT) using a previously published link to the Air‐N_2_ scale.^1^ The complete QCLAS and DI‐IRMS datasets for N_2_O RMs are shown in Tables S2 and S3 (supporting information).

**TABLE 7:** *δ* ^15^N^SP^ analyses of N_2_O RMs by QCLAS (Lab Empa) referenced to Air‐N_2_ by NH_4_NO_3_ decomposition as performed in this study (S1‐N_2_O/S4‐N_2_O) and by DI‐IRMS^1^ (Lab TT). All values are reported in ‰

	Lab Empa (QCLAS)		Lab TT (DI‐IRMS)		Difference *δ* ^15^N^SP^ (Lab Empa – Lab TT)	
	*δ* ^15^N^SP^	σ	*δ* ^15^N^SP^	σ		σ
**RM1A**	+0.47	0.26	−1.04	0.91	+1.51	0.95
**RM1B**	+0.30	0.30	−1.19	0.91	+1.49	0.96
**RM2**	+18.92	0.24	+17.00	0.91	+1.92	0.94
**RM3A**	−2.13	0.37	−4.13	0.93	+2.00	1.00
**RM3B**	+1.01	0.23	−0.68	0.91	+1.69	0.94
**RM4**	+0.00	0.60	−2.75	0.93	+2.75	1.11
**RM5**	+21.96	0.33	+20.20	0.91	+1.76	0.97

Results in Table 7 indicate a 1.5–2.7‰ offset in *δ* ^15^N^SP^ measurements by DI‐IRMS (Lab TT, Tokyo Institute of Technology) and QCLAS (Lab Empa) across all N_2_O RMs. This is most likely attributable to the calibration of the position‐dependent *δ* values with respect to Air‐N_2_ via the NH_4_NO_3_ decomposition technique, which were performed independently for the two labs. Incidentally, for the NH_4_NO_3_ salts S1–S4, the *δ* ^15^N(NO_3_^−^) results provided by Lab TT were always lower ((−0.63 ± 0.59)‰), while *δ* ^15^N(NH_4_^+^) values were higher than the respective weighted mean values ((+0.49 ± 0.25)‰), which would lead to 1.12‰ lower *δ* ^15^N^SP^ values (Table 5).

A similar 1.5–2.0‰ difference in *δ* ^15^N^SP^ results was recently detected by Kantnerová et al^54^ using an independent approach, equilibrating N_2_O at 200°C over a catalyst and comparing theoretical predictions with analytical results traceable to the *δ* ^15^N^SP^ scale of Lab TT. One previous comparison using an independent link to the Air‐N_2_ scale also indicated 1.5‰ higher *δ* ^15^N^SP^ values: (+20.2 ± 2.1)‰ vs. (+18.7 ± 2.2)‰ for ambient tropospheric N_2_O.^24^ Other studies confirmed the *δ* ^15^N^SP^ measurements by the Tokyo Institute of Technology, using the NH_4_NO_3_ decomposition technique.^23^, ^25^ The uncertainty of both approaches, however, was quite high.

We conclude that the realisation of the link between *δ* ^15^N^SP^ and the Air‐N_2_ scale with high accuracy is still challenging and the current realisation of the Air‐N_2_ scale for *δ* ^15^N^SP^ provided by USGS51 and USGS52^26^ may lead to too low *δ* ^15^N^SP^ values and should be revisited in future studies.

#### 3.2.2 Isotopic composition of N_2_O RMs for *δ* ^15^N by IRMS

*δ* ^15^N values of N_2_O RMs were analysed by IRMS in three different laboratories using independent links to the Air‐N_2_ scale (Table 8). Results display a consistent offset of (+0.22 ± 0.05)‰ and (+0.46 ± 0.14)‰ between Lab MPI‐I (EA/IRMS, Thermo Delta Plus, MPI‐BGC) and Lab UEA‐I (Sercon GEO 20‐20, UEA) versus Lab TT (Thermo MAT252, Tokyo Institute of Technology). A slightly larger offset was detected for the N_2_O RMs USGS51 and USGS52 (Table 9) by a comparison of published provisional values (Lab TT)^26^ and results of MPI‐II, with *δ* ^15^N values of (+0.92 ± 0.22)‰ (USGS51) and (+0.07 ± 0.22)‰ (USGS52). These values fall between results published by laboratories 7 and 8 (USGS and BGC‐IsoLab) in Ostrom et al,^26^ and are lower than results of the other laboratories, highlighting an ongoing scaling problem in *δ* ^15^N(N_2_O) measurements. A similar offset between laboratories had already been detected earlier and was attributed to differences in the propagation of the Air‐N_2_ scale to *δ* ^15^N(N_2_O).^20^, ^25^, ^26^ To account for differences between individual approaches to anchor laboratory results to scales, a weighted mean value was calculated for N_2_O RMs.

**TABLE 8:** *δ* ^15^N analyses of N_2_O RMs by IRMS at the Tokyo Institute of Technology (Lab TT: Thermo MAT252), MPI‐BGC (Lab MPI‐I: Thermo Delta Plus, Lab MPI‐II: Thermo MAT 253), and UEA (Lab UEA‐I: Sercon GEO 20‐20, Lab UEA‐II: Finnigan MAT 253) using independent calibration approaches. The ^17^O correction of DI‐IRMS was conducted using actual Δ^17^O values. All values are reported in ‰. The full set of analyses for all laboratories is provided in Table 9 (Lab MPI‐II) and in the supporting information (Lab TT: Table S3, Lab MPI‐I: Table S4, Lab UEA‐I: Table S5, Lab UEA‐II: Table S6)

	Lab TT	Lab MPI‐I	Lab MPI‐II	Lab UEA‐I	Lab UEA‐II	σ TT	σ MPI‐I	σ MPI‐II	σ UEA‐I	σ UEA‐II	Weighted mean ± σ
**RM1A**	+0.67	+0.44	+0.29	+0.29	+0.28	0.45	0.16	0.21	0.13	0.06	+**0.30 ± 0.05**
**RM1B**	+0.53	+0.33	+0.20	+0.24	+0.19	0.45	0.14	0.21	0.10	0.06	+**0.22 ± 0.05**
**RM2**	+7.31	+7.09	+6.95	+6.73	+6.94	0.45	0.16	0.21	0.07	0.06	+**6.88 ± 0.04**
**RM3A**	+53.41	+53.25	+53.11	+52.69	+53.09	0.47	0.15	0.21	0.11	0.07	+**53.02 ±** **0.05**
**RM3B**	+16.45	+16.14	+16.09	+15.96	+16.08	0.46	0.14	0.21	0.17	0.06	+**16.08 ±** **0.05**
**RM4**	+104.54	+104.39	+104.33	+104.18	+104.30	0.50	0.37	0.28	0.13	0.08	+**104.28 ±** **0.06**
**RM5**	+33.76	+33.52	+33.46	+33.38	+33.45	0.46	0.21	0.21	0.10	0.06	+**33.44 ±** **0.05**

**TABLE 9:** DI‐IRMS analyses of RMs, USGS51, USGS52 and NINO at MPI‐BGC (MPI‐II). Analyses were conducted in two campaigns in September 2019 and February 2021 on individual sample flasks. For RM1A, in each campaign three flask samples were analysed; for RM2, two flask samples were analysed in 2021. Referencing and ^17^O corrections considered actual Δ^17^O values: *δ* values were referenced to Air‐N_2_ and VSMOW using the in‐house working standard NINO (*δ* ^15^N) and USGS51 (*δ* ^18^O) and calculated according to Kaiser et al.^47^ *n* indicates the number of repeated analyses per campaign. Uncertainties for individual campaigns are calculated following the law of error propagation. For the uncertainty of the weighted mean, the uncertainty of the working standard was applied, which was considered as a conservative approach. All values are reported in ‰

	Sep 2019					Feb 2021					Weighted mean ± σ	
	*δ* ^15^N	σ	*δ* ^18^O	σ	n	*δ* ^15^N	σ	*δ* ^18^O	σ	n	*δ* ^15^N	*δ* ^18^O
RM1A	+0.30	0.22	+39.51	0.35	9	+0.29	0.23	+39.48	0.38	9	**+0.29 ± 0.21**	**+39.50 ± 0.34**
RM1B	+0.20	0.23	+39.14	0.36	3	+0.19	0.24	+39.10	0.38	3	**+0.20 ± 0.21**	**+39.12 ± 0.34**
RM2	+6.96	0.23	+44.37	0.35	3	+6.94	0.24	+44.32	0.38	6	**+6.95 ± 0.21**	**+44.35 ± 0.34**
RM3A	+53.12	0.25	+103.60	0.37	3	+53.09	0.26	+103.50	0.41	3	**+53.11 ± 0.21**	**+103.55 ± 0.34**
RM3B	+16.10	0.23	+55.58	0.35	3	+16.08	0.24	+55.55	0.39	3	**+16.09 ± 0.21**	**+55.57 ± 0.34**
RM4	+104.33	0.28	+154.93	0.38	3	n.a.	n.a.	n.a.	n.a.	n.a.	**+104.33 ± 0.21**	**+154.93 ± 0.38**
RM5	+33.48	0.23	+39.77	0.35	3	+33.44	0.25	+39.74	0.38	3	**+33.46 ± 0.21**	**+39.76 ± 0.34**
USGS51	+0.92	0.22	+41.45 ^a^	0.35 ^a^	6	n.a.	n.a.	n.a.	n.a.	n.a.	**+0.92 ± 0.22**	**+41.45 ± 0.35** ^a^
USGS52	+0.07	0.22	+40.89	0.35	6	n.a.	n.a.	n.a.	n.a.	n.a.	**+0.07 ± 0.22**	**+40.89 ± 0.35**
NINO	+0.54 ^b^	0.22 ^b^	+39.90	0.35	6	+0.53 ^c^	0.23 ^c^	+39.90 ^c^	0.38 ^c^	9	**+0.54 ± 0.21** ^b^	**+39.90 ± 0.34**

n.a. not analysed.

^a^ Average of laboratory results from Ostrom et al^26^ taken for referencing of *δ* ^18^O

^b^ Value provided by EA/IRMS analysis (Table S4, supporting information), value taken for referencing of *δ* ^15^N

^c^ Analysed as quality control.

In contrast, differences between analytical techniques applied within one lab, thus using the same link to the scale, were smaller than offsets between laboratories: (+0.10 ± 0.04)‰ for Lab MPI and (+0.12 ± 0.14)‰ for Lab UEA. This indicates that both EA/IRMS and GC‐IRMS and DI‐IRMS can achieve high accuracy, provided that an accurate link to the scale and Δ^17^O data are available. Consistency of N_2_O RM flask subsamples was demonstrated using DI‐IRMS (Lab MPI‐II, Table 9) by replicate sampling and analysis in two campaigns in September 2019 and February 2021. For RM1A, a total of six independent flask samples were analysed; the results agreed to within 0.02‰ for *δ* ^15^N(N_2_O) and 0.03‰ for *δ* ^18^O(N_2_O) (2σ, data not shown).

#### 3.2.3 Isotopic composition of N_2_O RMs for *δ* ^18^O and *δ* ^17^O by IRMS

*δ* ^18^O values of N_2_O RMs were analysed by IRMS in three different laboratories (Table 10). Results show deviations of (+0.30 ± 0.13)‰, (+0.22 ± 0.24)‰ and (+0.07 ± 0.38)‰ between Lab TT and Lab MPI‐II, Lab UEA‐I and Lab UEA‐II, respectively. Differences were highest for N_2_O RMs with high *δ* ^18^O values (RM3A, RM4), indicating a potential scaling or scale compression issue. Measurements were not completely independent for all laboratories, as the results for Lab MPI‐II were referenced to average *δ* ^18^O values of USGS51 in Ostrom et al,^26^ which in turn was determined by seven laboratories.

**TABLE 10:** *δ* ^18^O analyses of N_2_O RMs by IRMS at the Tokyo Institute of Technology (Lab TT: Thermo MAT252), MPI‐BGC (Lab MPI‐II: Thermo MAT 253), and UEA (Lab UEA‐I: Sercon GEO 20‐20, Lab UEA‐II: Finnigan MAT 253). All values are reported in ‰. The full set of analyses for all laboratories is provided in the supporting information (Lab TT: Table S3, Lab UEA‐I: Table S5)

	Lab TT	Lab MPI‐II	Lab UEA‐I	Lab UEA‐II	σ TT	σ MPI‐II	σ UEA‐I	σ UEA‐II	Weighted mean ± σ
**RM1A**	+39.37	+39.50	+39.06	+39.22	1.24	0.34	0.25	0.22	+**39.22 ±** **0.15**
**RM1B**	+38.86	+39.12	+38.77	+38.83	1.24	0.34	0.24	0.22	+**38.86 ±** **0.15**
**RM2**	+44.08	+44.35	+43.69	+44.02	1.25	0.34	0.24	0.22	+**43.96 ±** **0.15**
**RM3A**	+103.21	+103.55	+103.04	+102.78	1.30	0.34	0.27	0.24	+**103.04 ±** **0.16**
**RM3B**	+55.28	+55.57	+54.98	+55.13	1.26	0.34	0.26	0.22	+**55.17 ±** **0.15**
**RM4**	+154.35	+154.93	+155.17	+153.63	1.36	0.38	0.39	0.24	+**154.25 ±** **0.18**
**RM5**	+39.50	+39.76	+39.43	+39.50	1.29	0.34	0.24	0.22	+**39.52 ±** **0.15**

*δ* ^17^O values were determined by GC/IRMS at UEA (Lab UEA‐I) and showed a (0.98 ± 0.27)‰ offset to prototypical measurements by HR‐IRMS (MAT253 ULTRA) at the Tokyo Institute of Technology (Lab TT; Table 11). Consistency of GC/IRMS results agreed with an approximation, where the *δ* ^17^O was calculated from the ^17^O content of the ^18^O‐labelled H_2_O used for ^18^O‐labelled NH_4_NO_3_ and N_2_O production (certificate of analysis provided by Medical Isotopes Inc., USA; see Supplementary Method 2, supporting information). A 1‰ error in *δ* ^17^O results in around −0.1‰ error in *δ* ^15^N^α^, when used for correction of DI‐IRMS measurements.

**TABLE 11:** *δ* ^17^O analyses of N_2_O RMs by GC/IRMS at UEA (Lab UEA‐I), HR‐IRMS at the Tokyo Institute of Technology (Lab TT), and predictions based on mixing of ^18^O‐labelled N_2_O with commercial N_2_O. All values are reported in ‰

	Lab TT	Lab UEA‐I	Predicted	σ TT	σ UEA‐I
**RM1A**	+21.60	+20.33	+20.4	0.08	0.59
**RM1B**		+20.88	+20.2		0.56
**RM2**		+20.87	+20.8		0.40
**RM3A**	+24.40	+23.78	+24.5	0.21	0.54
**RM3B**		+21.22	+21.5		0.24
**RM4**	+27.75	+26.71	+27.6	0.35	0.83
**RM5**		+20.90	+20.6		0.44

## 4 CONCLUSIONS

Within the SIRS project, we established seven pure N_2_O isotope RMs, which were analysed by specialised laboratories against the international isotope‐ratio scales. The established N_2_O isotope RMs offer a wide coverage of *δ* values (Table 12) beyond the currently available standards USGS51 and USGS52. This will enable future users to implement the recommended two‐point calibration approach for IRMS instrumentation, and, upon dilution with an appropriate gas matrix, for laser spectroscopic techniques as well.^19^, ^55^ In addition, the gases have been characterised for their *δ* ^17^O signatures in order to improve data quality/correction algorithms with respect to *δ* ^15^N^SP^ and *δ* ^15^N analysis by mass spectrometry. In summary, the novel N_2_O isotope RMs are expected to improve compatibility between laboratories and accelerate the progress in this emerging field of research.

**TABLE 12:** Weighted mean *δ* values for the N_2_O RMs. All values are reported in ‰

	*δ* ^15^N^SP^	σ	*δ* ^15^N	σ	*δ* ^18^O	σ	*δ* ^17^O	σ
**RM1A**	−1.04	0.91	+0.30	0.05	+39.22	0.15	+20.33	0.59
**RM1B**	−1.19	0.91	+0.22	0.05	+38.86	0.15	+20.88	0.56
**RM2**	+17.00	0.91	+6.88	0.04	+43.96	0.15	+20.87	0.40
**RM3A**	−4.13	0.93	+53.02	0.05	+103.04	0.16	+23.78	0.54
**RM3B**	−0.68	0.91	+16.08	0.05	+55.17	0.15	+21.22	0.24
**RM4**	−2.75	0.93	+104.28	0.06	+154.25	0.18	+26.71	0.83
**RM5**	+20.20	0.91	+33.44	0.05	+39.52	0.15	+20.90	0.44

### PEER REVIEW

The peer review history for this article is available at https://publons.com/publon/10.1002/rcm.9296.

## Supporting information


**Table S1.** Minimum, maximum, and average reaction yield for NH_4_NO_3_ thermal decomposition at 270°C for salts S1 – S6. S1* indicates the decomposition of S1 in a NH_4_HSO_4_‐(NH_4_)_2_SO_4_ melt. *n* indicates the number of decomposition experiments.
**Table S2.** Isotopic composition of RMs, analysed by QCLAS at Empa versus S1‐N_2_O and S4‐N_2_O to calculate *δ* ^15^N^α^, *δ* ^15^N^β^, *δ* ^15^N^SP^, and *δ* ^15^N values. *n* indicates the number of analyses. Uncertainties are calculated using the law of error propagation involving the uncertainties in S1‐N_2_O and S4‐N_2_O, their analyses, and the analyses of N_2_O RMs but do not enclose deviations due to fractionation or branching effects during NH_4_NO_3_ decomposition. All values are reported in ‰.
**Table S3.** Isotopic composition of RMs, analysed by IRMS at Tokyo Institute of Technology (Lab TT) versus an isotopically characterised in‐house working standard to calculate *δ* ^15^N^α^, *δ* ^15^N^β^, *δ* ^15^N^SP^, *δ* ^15^N, and *δ* ^18^O values on the “Tokyo Tech scale”. *n* indicates the number of analyses. Uncertainties are calculated using the law of error propagation. All values are reported in ‰.
**Table S4.** *δ* ^15^N of RMs, the in‐house N_2_O standard gas (NINO), and a number of quality control standards, analysed by Lab MPI (EA‐IRMS, Thermo Delta plus, MPI‐I) versus primary reference materials and second scale anchor of the Air‐N_2_ scale (IAEA‐N1, USGS32). *n* indicates the number of analyses. Expanded uncertainties are calculated following the law of error propagation. For the quality control, standards target values and references are provided as well. All values are reported in ‰.
**Table S5.** Isotopic composition of RMs, analysed as N_2_O diluted to 0.09 mmol mol^−1^ on Sercon GEO 20–20 IRMS (UEA‐I) after gold decomposition, scale‐normalised to the *δ* ^18^O value of RM4. *n* indicates the number of analyses. Uncertainties are calculated using the law of error propagation from the standard deviations of replicate measurements against the working reference gas and the calibration uncertainties of the working reference gas against Air‐N_2_ and VSMOW.^14^ All values are reported in ‰.
**Table S6.** Isotopic composition of RMs, analysed as pure N_2_O on Finnigan MAT 253 IRMS (UEA‐II) using the actual *Δ* ^17^O measurements. *n* indicates the number of analyses. Uncertainties are calculated using the law of error propagation from the standard deviations of replicate measurements against the working reference gas and the calibration uncertainties of the working reference gas against Air‐N_2_ and VSMOW.^15^ All values are reported in ‰.

## Data Availability

The data that supports the findings of this study are available in the supplementary material of this article. Further data are available from the corresponding author upon reasonable request.
