# Supplementary material for: Isotopically characterised N2O reference materials for use as community standards
Source: Rapid Commun Mass Spectrom. 2022 Apr 28;36(13):e9296. doi: 10.1002/rcm.9296 (PMC9286586; doi:10.1002/rcm.9296)
Supplement: Supplementary file 1 — Table S1. Minimum, maximum, and average reaction yield for NH4NO3 thermal decomposition at 270°C for salts S1 – S6. S1* indicates the decomposition of S1 in a NH4HSO4‐(NH4)2SO4 melt. n indicates the number of decomposition experiments. Table S2. Isotopic composition of RMs, analysed by QCLAS at Empa versus S1‐N2O and S4‐N2O to calculate δ 15Nα, δ 15Nβ, δ 15NSP, and δ 15N values. n indicates the number of analyses. Uncertainties are calculated using the law of error propagation involving the uncertainties in S1‐N2O and S4‐N2O, their analyses, and the analyses of N2O RMs but do not enclose deviations due to fractionation or branching effects during NH4NO3 decomposition. All values are reported in ‰. Table S3. Isotopic composition of RMs, analysed by IRMS at Tokyo Institute of Technology (Lab TT) versus an isotopically characterised in‐house working standard to calculate δ 15Nα, δ 15Nβ, δ 15NSP, δ 15N, and δ 18O values on the “Tokyo Tech scale”. n indicates the number of analyses. Uncertainties are calculated using the law of error propagation. All values are reported in ‰. Table S4. δ 15N of RMs, the in‐house N2O standard gas (NINO), and a number of quality control standards, analysed by Lab MPI (EA‐IRMS, Thermo Delta plus, MPI‐I) versus primary reference materials and second scale anchor of the Air‐N2 scale (IAEA‐N1, USGS32). n indicates the number of analyses. Expanded uncertainties are calculated following the law of error propagation. For the quality control, standards target values and references are provided as well. All values are reported in ‰. Table S5. Isotopic composition of RMs, analysed as N2O diluted to 0.09 mmol mol−1 on Sercon GEO 20–20 IRMS (UEA‐I) after gold decomposition, scale‐normalised to the δ 18O value of RM4. n indicates the number of analyses. Uncertainties are calculated using the law of error propagation from the standard deviations of replicate measurements against the working reference gas and the calibration uncertainties of the working [file RCM-36-0-s001.docx]

# Supporting information

# Isotopically-characterised N_2_O reference materials for use as community standards

Joachim Mohn^1^, Christina Biasi^2^, Samuel Bodé^3^, Pascal Boeckx^3^, Paul J. Brewer^4^, Sarah Eggleston^1,^^, Heike Geilmann^5^, Myriam Guillevic^1,^°, Jan Kaiser^6^, Kristýna Kantnerová^1,§^, Heiko Moossen^5^, Joanna Müller^1,*^, Mayuko Nakagawa^7^, Ruth Pearce^4^, Isabell von Rein^5^, David Steger^1^, Sakae Toyoda^8^, Wolfgang Wanek^9^, Sarah K. Wexler^6^, Naohiro Yoshida^7,8^ & Longfei Yu^1,+^

^1^Laboratory for Air Pollution / Environmental Technology, Empa, 8600 Dübendorf, Switzerland

^2^Department of Environmental and Biological Sciences, University of Eastern Finland, Kuopio, Finland

^3^Isotope Bioscience Laboratory – ISOFYS, Department of Green Chemistry and Technology, Faculty of Bioscience Engineering, Ghent University, Gent, Belgium

^4^National Physical Laboratory, Teddington, Middlesex TW11 0LW, United Kingdom

^5^Beutenberg Campus, Max‐Planck‐Institute for Biogeochemistry, 07701 Jena, Germany

^6^Centre for Ocean and Atmospheric Sciences, School of Environmental Sciences, University of East Anglia, Norwich, UK

^7^Earth‐Life Science Institute, Tokyo Institute of Technology, Tokyo 152‐8550, Japan

^8^Department of Chemical Science and Engineering, School of Materials and Chemical Technology, Tokyo Institute of Technology, Yokohama 226‐8502, Japan

^9^Terrestrial Ecosystem Research, Centre for Microbiology and Environmental Systems Science, University of Vienna, Vienna, Austria

^ now at: PAGES International Project Office, 3012 Bern, Switzerland

° now at: Air Pollution Control and Chemicals Division, Federal Office for the Environment, 3003 Bern, Switzerland

§ now at: Thermo Fisher Scientific, 28199 Bremen, Germany

* now at: Plant Protection Chemistry, Agroscope, 8820 Wädenswil, Switzerland

+ now at: Department of Ecology, College of Life Science and Technology, Jinan University, Guangzhou, 510632, PR China

Correspondence: J. Mohn, Laboratory for Air Pollution / Environmental Technology, Empa, Überlandstr. 129, CH‐8600, Dübendorf, Switzerland. Email: joachim.mohn@empa.ch

## Supplementary Method 1: Analytical techniques used by participating laboratories for *δ*^15^N(NH_4_^+^), *δ*^15^N(NO_3_^-^), and *δ*^15^N(NH_4_NO_3_) analyses

**Institution: MPI-BGC (Lab (1))**

*δ*^15^N(NH_4_NO_3_)

*δ*^15^N(NH_4_NO_3_) analysis was conducted using an elemental analyzer (EA 1100, CE, Rodano, Italy) coupled to an IRMS (Delta Plus, Thermo Finnigan, Bremen, Germany). Samples and standards were weighed into tin capsules (*n* = 5), each containing 100 µg of nitrogen. International standards: IAEA-N-1 and USGS32 were used to realize the Air-N_2_ scale and to correct for scale contraction. A third primary standard and in-house standards were used as quality control standards. Note that USGS32 does not have an assigned uncertainty, as the given value is a consensus value. The salts were analyzed multiple times over a two-month period. IAEA-N-2 was analyzed in each daily sequence as a quality control measurement. The average measured value for IAEA-N2 over the measurement period was (+20.41 ± 0.06) ‰.

**Institution: UC Davis (Lab (2))**

*δ*^15^N(NH_4_NO_3_)

Samples (0.15 mg; *n* = 3) were analysed by EA-IRMS (Elementar Vario EL Cube, Elementar, Langenselbold, Germany) interfaced to an Elementar VisION IRMS (Elementar UK Ltd, Cheadle, UK). Addition of oxygen for either 40 s, 50 s, 70 s, or 80 s did not affect the results. Measurements were calibrated against internal standards glutamic acid (*δ*^15^N = (−6.80 ± 0.08) ‰), nylon6 (*δ*^15^N = (−10.54 ± 0.07) ‰), enriched alanine (*δ*^15^N = (+41.13 ± 0.07) ‰), and bovine liver (*δ*^15^N = (+7.70 ± 0.09) ‰), and corrected against USGS25, USGS26, IAEA-N-1, IAEA-N-2, USGS32, USGS34, and USGS35.

**Institution: University of Ghent (Lab (3))**

*δ*^15^N(NH_4_^+^)

Samples were diluted with ultrapure water to 15 µM, 4 mL of this solution was transferred to a 20 mL headspace vial. To this headspace vial, 0.4 mL of a hypobromite (BrO^−^) solution was added to oxidise the NH_4_^+^ to NO_2_^−^. [The BrO^−^ solution was prepared freshly by adding 3 mL of 6 M HCl to 50 mL of a solution containing 2.4 mg of NaBrO_3_ and 20 mg of NaBr in the dark; after 5 min, 50 mL of 10 M NaOH was added to produce BrO^−^. After 30 min, 0.05 mL of NaAsO_2_ solution (0.4 M) was added to remove excess BrO^−^.] After the addition of 0.5 mL of 6 M HCl and 0.5 mL NH_2_OH·HCl solution (0.24 mM), the headspace vials were closed and kept overnight at 37 °C. Finally, 0.5 mL of 5 M NaOH was added through the septum to stop the reaction and reduce the CO_2_ concentration in the headspace. The isotopic ratio of the resulting N_2_O was analysed by Trace Gas - Isotope Ratio Mass Spectrometer (TG-IRMS, ANCA-TGII interfaced with a SerCon 20-20 IRMS, all SysCon electronics). Samples (*n* = 5) were calibrated against USGS25, USGS26, IAEA-N-1, and IAEA-N-2.^1^

*δ*^15^N(NO_3_^−^)

Analysis of *δ*^15^N(NO_3_^−^) was conducted based on the quantitative transformation of NO_3_^−^ to N_2_O by a denitrifying bacteria lacking the gene for N_2_O reductase (*Pseudomonas aureofaciens*). Samples were diluted to 35 µM in ultrapure water, and 5 mL was transferred through a septum to a 20 mL headspace vial containing 2 mL of concentrated *Pseudomonas* slurry previously purged for 3 h with N_2_. Vials were incubated overnight at 25 °C. The reaction was stopped by adding 10 M NaOH, which also reduced the CO_2_ concentration of the headspace. The isotopic composition of the formed N_2_O was determined using TG-IRMS as described for *δ*^15^N(NH_4_^+^). Samples (*n*= 5) were calibrated against USGS34, USGS32, and USGS35.^adapted from2,3^

*δ*^15^N(NH_4_NO_3_)

Samples (*n* = 6) and standards containing approx. 10 µmol N were weighed into tin capsules and analysed by EA-IRMS (ANCA-SL interfaced with a SerCon 20-22 IRMS, SysCon electronics). No oxygen pulse was added to the high-temperature reactor. Normalisation on the Air-N_2_ scale was done using USGS25, USGS34, USGS26, USGS32, IAEA-N-1, IAEA-N-2, and USGS35.^4^

**Institution: University of Pittsburgh (Lab (4))**

*δ*^15^N(NO_3_^−^)

For the measurement of *δ*^15^N(NO_3_^−^), 20 nmol of NH_4_NO_3_ (*n* = 3 – 5) were transformed into N_2_O by the bacterial denitrifier *Pseudomonas auerofaciens*. N_2_O was introduced to IRMS (IsoPrime, Elementar, Langenselbold, Germany) interfaced with a trace gas pre-concentrator (Trace Gas, Elementar, Langenselbold, Germany). Measurements were calibrated using international *δ*^15^N reference standards for NO_3_^−^: IAEA-NO-3, USGS32, and USGS34, analysed following the IT principle. The analytical error for *δ*^15^N(NO_3_^−^) is ± 0.2 ‰.^2,3^

*δ*^15^N(NH_4_NO_3_)

1 mL of a bromate/bromide stock solution (0.6 g of NaBrO_3_ and 5 g of NaBr dissolved in 250 mL of deionized water) was added to 50 mL of Milli-Q water and 3 mL of 6 M HCl (BrO working solution). The mixture was allowed to react in the dark for 5 min and 50 mL of 10 N NaOH was added to the solution. NH_4_^+^ samples were diluted to 10 μmol of NH_4_^+^ and 20 mL of the sample was transferred to acid-washed 50 mL centrifuge vials. To oxidize sample NH_4_^+^ to NO_2_^−^, 2 mL of the BrO working solution was added to the sample vial. The vials were immediately capped and hand-shaken vigorously for 30 s followed by shaking for 1 h on a shaker table. After oxidation, the sample pH was adjusted to between 3 and 9 using 6 N HCl. If the pH was below 3, it was readjusted to the 3 to 9 range using 10 N NaOH. 20 nmol of sample NO_2_^−^ was then converted into N_2_O using the bacterial denitrifier *Pseudomonas aureofaciens* and introduced into an isotope-ratio mass spectrometer (Elementar IsoPrime, Langenselbold, Germany) equipped with a trace gas pre-concentrator (Trace Gas, Elementar, Langenselbold, Germany). Measurements (*n* = 4-5) were calibrated against the following international standards: IAEA-N-1, IAEA-N-2, IAEA-NO-3, USGS25, USGS26, USGS32, and USGS34 adhering to the IT principle.^5^

**Institution: UEF-BGC (Lab (5))**

*δ*^15^N(NH_4_^+^)

For *δ*^15^N(NH_4_^+^) analysis by microdiffusion, samples were dissolved in 1 M KCl to reach a final concentration of 100 μM, treated with MgO and incubated with acid traps at 37 °C for 3 days. Acid traps were pieces of glass-fibre filter impregnated with KHSO_4_ and covered with Teflon tape. After the incubation period, the acid traps were collected and dried under reduced pressure over concentrated H_2_SO_4_. Thereafter, the acid traps were introduced to an elemental analyser (Flash EA 1112 Series, Thermo Finnigan, Bremen, Germany) coupled to an IRMS (Delta Plus XP, Thermo Fisher, Bremen, Germany) at the University of Eastern Finland and analysed for *δ*^15^N. Each sample was measured 4-5 times. Results were calibrated using the following laboratory standards: prolin (*δ*^15^N = (−8.87 ± 0.13) ‰) and wheat (*δ*^15^N = (+3.32 ± 0.16) ‰) using a two-point calibration approach. In addition, results were corrected using the following standards: USGS25, USGS26, IAEA-N-1, IAEA-N-2. All laboratory and quality control standards were prepared in the same matrix and processed according to the identical treatment (IT) principle.^6^

*δ*^15^N(NO_3_^−^)

For *δ*^15^N(NO_3_^−^) analysis by chemical method, samples were dissolved in 1 M KCl to reach a final concentration of 100 μM. An aliquot of 400 μL sodium azide buffer (2.6 g sodium azide dissolved in 20 mL MQ water (2 M) and mixed with 20 mL 20% acetic acid solution) and 2.5 mL VCl_3_ solution (2.4 g VCl_3_ dissolved in 300 mL 1 M HCl) were added and the mixture reacted at 37 °C for 18 h. 10 M NaOH was then added to stop the reaction. Immediately after termination, the head space gas was analysed using a headspace gas sampler (Gas-Bench II, Thermo Fisher, Bremen, Germany) coupled to an IRMS (Delta V Advantage, Thermo Fisher, Bremen, Germany) at the University of Vienna. Each sample was measured 4-5 times. Results were calibrated by linear regression equation to correct for N_2_O reference gas offset and azide contribution using the following laboratory standards: KNO_3_ (*δ*^15^N = (−2.8 ± 0.05) ‰), KNO_3_ (*δ*^15^N = (+5.3 ± 0.08) ‰), Ca(NO_3_)_2_ x 4 H_2_O (*δ*^15^N = (+9.3 ± 0.06) ‰), and AgNO_3_ (*δ*^15^N = (+16.8 ± 0.27) ‰). In addition, results were corrected using the following standards: USGS32, USGS34, USGS35. All standards were prepared in the same matrix as the samples.^6^

**Institution: University of Vienna (Lab (6))**

*δ*^15^N(NH_4_^+^)

For *δ*^15^N(NH_4_^+^) analysis by microdiffusion (*n* = 4), 40.02 mg of NH_4_NO_3_ and 33.04 mg of (NH_4_)_2_SO_4_ were dissolved in 50 mL of Milli-Q water separately as stock solutions (N concentration was 10 mM) and diluted with 1 M KCl solution to an N concentration of 0.4 mM. 10 mL of the solution was supplemented with 100 mg of MgO and one acid trap. Acid traps were prepared as described above (UEF). After incubation for 5 days on a shaker at room temperature, the acid traps were removed, dried over concentrated H_2_SO_4_ in a desiccator, and analyzed by EA-IRMS, an elemental analyzer (Carlo Erba 1110, CE Instruments, Italy) coupled to a Delta^Plus^ isotope-ratio mass spectrometer (Finnigan MAT, Germany) via a Conflo III interface (Thermo Fisher, Austria). Analytical results were calibrated using the following laboratory standard: PSS7 (*δ*^15^N = (+0.11 ± 0.08) ‰). In addition, a number of certified quality control standards and blanks were analyzed: USGS25, USGS 26, IAEA-N-1, and IAEA-N-2 and used for correction. Treatment of all standards and blanks followed the IT principle.^6^

*δ*^15^N(NO_3_^−^)

40.02 mg of NH_4_NO_3_, 50.55 mg of KNO_3_, and 42.50 mg of NaNO_3_ were separately dissolved in 50 mL of Milli-Q water at a concentration of 10 mM and diluted with Milli-Q water to 50 μM. Sodium azide buffer and VCl_3_ solution were added, and the mixture was incubated on a shaker at 37 °C for 24 h. Afterwards, 6 M NaOH was added to stop the reaction and the produced N_2_O analysed by a purge-and-trap IRMS (PT-IRMS). The PT-IRMS system consisted of a cryofocusing unit of a Gasbench II headspace analyzer (Thermo Fisher, Bremen, Germany) coupled to a Finnigan Delta V Advantage IRMS (Thermo Fisher, Bremen, Germany). Analytical results (*n* = 4) were calibrated using the following laboratory standards: KNO_3_ (Riedel deHaën 31263, ≥ 99%, Lot# 1247A; *δ*^15^N = (−2.8 ± 0.05) ‰); KNO_3_ (Fluka 60419, p.A., Lot# 428317/1; *δ*^15^N = (5.3 ± 0.08) ‰), AgNO_3_ (Merck 1512, ACS-ISO Reag. Ph Eur, Lot# 8562371; *δ*^15^N = (+16.8 ± 0.27) ‰), using a linear regression approach. In addition, a number of certified quality control standards: USGS32, USGS34 and USGS35 and blanks (Milli-Q water) were analysed and used for correction. All standards and blanks were treated according to the IT principle.^6^

**Institution: Tokyo Institute of Technology (Lab (7))**

*δ*^15^N(NH_4_^+^)

16 mg (200 μmol) of NH_4_NO_3_ was distilled with water vapor after 10 mL of 10 M KOH solution was added. The distilled ammonia was absorbed into 10 mL of 0.25 M H_2_SO_4_ solution until the total amount of the solution became about 30 mL. The ammonium ion solution was concentrated at about 80 °C on a hot plate to reduce the amount into approx. 10 mL and oxidized to N_2_ by adding 5 mL of KBrO (0.8 M, prepared by addition of Br_2_ to KOH solution) on a vacuum line. The isotope ratio of the N_2_ collected on molecular sieves 5 Å at liquid nitrogen temperature was determined on a mass spectrometer (MAT 253, Thermo Fisher Scientific, Bremen, Germany). Results were corrected using the following standards, USGS25, USGS26, IAEA-N-1, and IAEA-N-2, according to the IT principle. All measurements were done in triplicate in three individual runs. Within each measurement run, in 8 cycles, sample N_2_ was analysed against reference N_2_.^7^

*δ*^15^N(NO_3_^−^)

After the above, 0.5 g of Devarda’s alloy was added to the residual solution, and NO_3_^−^ was reduced to NH_4_^+^ at about 70 °C. After the reaction was complete, the resulting NH_3_ was collected and converted in the same manner as described above. Results were corrected using the following standards, USGS32, USGS34, and USGS35, according to the IT principle. All measurements were done in triplicate in three individual runs. Within each measurement run, in 8 cycles, sample N_2_ was analysed against reference N_2_.^7^

**Institution: Hydroisotop (Lab (8))**

*δ*^15^N(NH__^+^)

5 mg of sample was dissolved in 10 mL of distilled water and added to a compartment of a 150 mL reactor, while 10 mL of 15 M LiBrO solution were added to a second compartment. After the reaction vessel was evacuated, the two solutions were mixed and the thereby formed nitrogen gas was analysed by IRMS (Delta V Plus, Thermo Scientific, Bremen, Germany). Each sample was measured two or three times. Sample gas measurements were calibrated using laboratory standards: N_2_ (*δ*^15^N = (−1.4 ± 0.3) ‰), LS 1 (NH_4_)_2_SO_4_ (*δ*^15^N = (−2.7 ± 0.4) ‰, LS 2 (NH_4_)_2_SO_4_ (*δ*^15^N = (+17.0 ± 0.3) ‰) previously analysed against international standards (IAEA-N-2). In addition, a number of certified quality control standards were analysed: USGS25, USGS26, IAEA-N-1, and IAEA-N-2, and used for correction. The analytical error for *δ*^15^N(NH_4_^+^) is ± 0.3 ‰ based on *δ*^15^N_AIR-N2_.

*δ*^15^N(NO_3_^−^)

20 mg of sample was dissolved in 20 mL of water and the cation (NH_4_^+^) of the diluted sample was removed by ionic exchange (ion-exchange column filled with IR-120 resin) against potassium. After removal of the solvent, the aliquot of residue (300 μg) was weighed into Ag-capsules and measured by EA (Flash 200, Thermo Scientific, Bremen, Germany) coupled to IRMS (Delta V Plus, Thermo Scientific, Bremen, Germany). Each sample was measured two or three times and calibrated against laboratory standards previously analysed against international standards: IAEA-NO-3, USGS34, and USGS32. In addition, a number of certified quality control standards: USGS32, USGS34, and USGS35, were analysed and used for correction. The analytical error for *δ*^15^N(NO_3_^−^) is ± 0.3 ‰ based on *δ*^15^N_AIR-N2_.^8-10^

## Supplementary Method 2: Production and analysis of ^18^O-enriched N_2_O and ^15^N^β^-depleted N_2_O

Synthesis of ^18^O-enriched N_2_O:

Due to very high prices for custom synthesis, NN^18^O was synthesized in-house using the following procedure: (i) ^18^O-exchange of HNO_3_ (1.8 mL; Sigma Aldrich, Buchs, Switzerland) with 97% H_2_^18^O (5 mL; analysis: 97.7% H_2_^18^O, 1.2% H_2_^17^O, 1.1% H_2_^16^O; Medical Isotopes Inc., Pelham, USA) under reflux for 24 h; (ii) condensation of NH_3_ and reaction controlled by LN_2_, drying of product NH_4_NO_3_; (iii) thermal decomposition of NH_4_NO_3_ in batches of 1 g in 150 mL glass bulbs with break-seal to produce 200 mL of N_2_O in each batch (yield approx. 70%). (i) and (ii) were conducted on a vacuum line under N_2_ atmosphere (N_2_ dried over P_2_O_5_) to avoid contamination with ^16^O from air moisture (H_2_O). The total volume of produced NN^18^O gas was 725 mL.

Analysis of ^18^O-enriched N_2_O:

The isotopic enrichment in NN^18^O versus NN^16^O was analyzed with a quadrupole mass spectrometer (QMS) equipped with a customized ambient-pressure inlet system (Vision 1000C, MKS Instruments, UK). The following QMS settings were selected: mass range *m*/*z* 11 – 50, accuracy 8, electronic gain 20k, detector multiplier 2.222 x 10^-8^, filament 1. Prior to the analysis, the pure ^18^O‑enriched N_2_O was diluted with N_2_ (99.9999%, Messer Schweiz AG, Lenzburg, Switzerland) to a target concentration of 3400 μmol mol^-1^ N_2_O using a ten-port two-position valve (EH2C10WEPH, Valco Instruments Inc., Schenkon, Switzerland) with a 3-mL sample loop into a 1800 mL stainless-steel cylinder (BRC Rasmussen, Portland, USA). Measurements were referenced to 5001 μmol mol^-1^ N_2_O in N_2_ (2% uncertainty, Messer Schweiz AG, Lenzburg, Switzerland) dynamically diluted with N_2_ (99.9999%, Messer Schweiz AG, Lenzburg, Switzerland) to 1000, 2000, 3000, 4000, 5001 μmol mol^-1^ N_2_O using mass flow control devices (Vögtlin Instruments GmbH, Muttenz, Switzerland).

Triplicate analysis of ^18^O-enriched N_2_O resulted in the following composition: (36.25 ± 0.10)% NN^16^O and (63.75 ± 0.76)% NN^18^O. The absence of CO_2_ impurities in ^18^O-enriched N_2_O was assured by implementing an Ascarite trap with no significant difference detected. The sum of ^17^O (^14^N^14^N^17^O) and ^15^N (^15^N^14^N^16^O and ^14^N^15^N^16^O) analyzed as *m*/*z* 45/44 was (8.1 ± 1.0) ‰, which is in agreement with the natural ^15^N abundance and the ^17^O content of H_2_^18^O applied for the ^18^O-exchange.

Synthesis of ^15^N^β^-depleted N_2_O:

As no commercial source for ^15^N^β^-depleted N_2_O was available, NH_4_NO_3_ salt that was ^15^N-depleted in NH_4_^+^ (0.306% ^15^NH_4_^+^; Shoko Science Co., Ltd., Japan), obtained from Sakae Toyoda (Tokyo Institute of Technology), was thermally decomposed as described above for NN^18^O. According to the manufacturer, specification of *δ*^15^N^β^-N_2_O of −160 ‰ was expected.

Analysis of ^15^N^β^-depleted N_2_O:

The ^15^N-depletion in ^15^N^β^-depleted N_2_O was analyzed with the QCLAS spectrometer (Aerodyne Research Inc., USA) described in 3.2.4. Prior to the analysis, the pure ^15^N^β^-depleted N_2_O was diluted with synthetic air (20.5% O_2_ in N_2_, 99.999%, Messer Schweiz AG, Lenzburg, Switzerland) to a target concentration of 95 – 100 μmol mol^-1^ using a ten-port two-position valve (EH2C10WEPH, Valco Instruments Inc., Schenkon, Switzerland) with 1 mL sample loop into a 6000 mL stainless-steel cylinder (S6L Series, LabCommerce Inc., San Jose, USA). Measurements were referenced to laboratory standards previously analyzed by Sakae Toyoda (Tokyo Institute of Technology). Triplicate analysis provided the following isotopic composition: *δ*^15^N^α^ = (−2.537 ± 0.005) ‰, *δ*^15^N^β^ = (−162.211 ± 0.032) ‰, *δ*^18^O = (+38.915 ± 0.003) ‰.

## Supplementary Method 3: Analysis of N_2_O RMs for *δ*^17^O by HR-IRMS at Tokyo Institute of Technology (Lab TT)

The measurement of N_2_O^+^ molecule ions, including ^14^N_2_^16^O^+^, ^14^N_2_^17^O^+^, and ^14^N_2_^18^O^+^, was performed at a mass resolution of 1.6 – 1.9 × 10^4^ using a 16-μm slit (middle resolution). For each set of measurements, the detectors were positioned so that the flat tops of every peak of interest were aligned. All measurements were made in the dual-inlet mode, and every sample and standard intensity comparison (‘acquisition’) was composed of 8 cycles with 8 s integration time for each individual measurement. The measurement was performed at 9.9 kV accelerating voltage, a filament emission current of 1.65 mA, and an equilibration time of 35 s. Before each acquisition cycle, a series of 500 s background scans were collected for both sample and reference gases. Six acquisitions were collected and standard errors were estimated.The *m*/*z* 44, 45, and 46 ion currents were detected by Faraday cups with 10^10^ Ω, 10^12^ Ω, and 10^12^ Ω amplifiers, respectively. *δ*^17^O measurements were conducted against a laboratory N_2_O working gas (YC-502; *δ*^18^O = (+20.63 ± 0.06) ‰ and an *δ*^17^O =(+10.84 ± 0.03) ‰, based on a mass-dependent relationship between ^18^O and ^17^O with the slope of 0.528). *δ*^17^O of RM3A and RM4 were calibrated using the relative differences from RM1A *δ*^17^O value.

## Table S1

| Table S1. Minimum, maximum, and average reaction yield for NH_4_NO_3_ thermal decomposition at 270 °C for salts S1 – S6. S1* indicates the decomposition of S1 in a NH_4_HSO_4_-(NH_4_)_2_SO_4_ melt. *n* indicates the number of decomposition experiments. | | | | |
| --- | --- | --- | --- | --- |
|  | yield (%) | | | *n* |
|  | min | max | avg ± σ |  |
| S1 | 94.3 | 94.6 | 94.4 ± 0.1 | 4 |
| S1* | 95.7 | 97.5 | 96.7 ± 0.6 | 7 |
| S2 | 95.1 | 95.2 | 95.2 ± 0.1 | 4 |
| S3 | 94.8 | 95.5 | 95.1 ± 0.3 | 4 |
| S4 | 94.8 | 96.0 | 95.3 ± 0.5 | 6 |
| S5 | 94.9 | 95.6 | 95.2 ± 0.3 | 4 |
| S6 | 91.9 | 93.8 | 92.6 ± 0.6 | 5 |

## Table S2

In the main manuscript (section 3.2), QCLAS results for *δ*^15^N^SP^_cal_ are compared with results provided by DI-IRMS (Tokyo Institute of Technology) using an independent link to the Air-N_2_ scale. For *δ*^15^N, no comparison of QCLAS results to results provided by other laboratories was conducted as the provided link through *δ*^15^N(NO_3_^−^) and *δ*^15^N(NH_4_^+^) analysis is subject to substantially higher uncertainties. The observed systematic decreasing trend in *δ*^15^N values of around 0.5 ‰ from low (RM1) to high (RM4) *δ* values, compared to IRMS results of Tokyo Institute of Technology, may be at least partly caused by a systematic trend in *δ*^15^N(NH_4_^+^) values of S1 and S4 used for normalization, while the difference in *δ*^15^N^α^ was consistent throughout the data set.

| Table S2. Isotopic composition of RMs, analysed by QCLAS at Empa versus S1-N_2_O and S4-N_2_O to calculate *δ*^15^N^α^, *δ*^15^N^β^, *δ*^15^N^SP^, and *δ*^15^N values. *n* indicates the number of analyses. Uncertainties are calculated using the law of error propagation involving the uncertainties in S1-N_2_O and S4-N_2_O, their analyses, and the analyses of N_2_O RMs but do not enclose deviations due to fractionation or branching effects during NH_4_NO_3_ decomposition. All values are reported in ‰. | | | | | | | | | |
| --- | --- | --- | --- | --- | --- | --- | --- | --- | --- |
|  | *δ*^15^N^α^ |  | *δ*^15^N^β^ |  | *δ*^15^N^SP^ |  | *δ*^15^N |  | *n* |
| RM1A | +1.13 | 0.19 | +0.62 | 0.18 | +0.47 | 0.26 | +0.88 | 0.26 | 6 |
| RM1B | +0.81 | 0.21 | +0.51 | 0.21 | +0.30 | 0.30 | +0.66 | 0.30 | 7 |
| RM2 | +16.84 | 0.19 | −2.11 | 0.14 | +18.92 | 0.24 | +7.36 | 0.24 | 7 |
| RM3A | +52.24 | 0.29 | +54.37 | 0.23 | −2.13 | 0.37 | +53.30 | 0.37 | 7 |
| RM3B | +17.40 | 0.19 | +16.40 | 0.14 | +1.01 | 0.23 | +16.90 | 0.23 | 7 |
| RM4 | +104.20 | 0.46 | +104.20 | 0.40 | +0.00 | 0.60 | +104.20 | 0.60 | 8 |
| RM5 | +44.98 | 0.29 | +23.02 | 0.16 | +21.96 | 0.33 | +34.00 | 0.33 | 7 |

### Table S3

| Table S3. Isotopic composition of RMs, analysed by IRMS at Tokyo Institute of Technology (Lab TT) versus an isotopically characterised in-house working standard to calculate *δ*^15^N^α^, *δ*^15^N^β^, *δ*^15^N^SP^, *δ*^15^N, and *δ*^18^O values on the "Tokyo Tech scale". *n* indicates the number of analyses. Uncertainties are calculated using the law of error propagation. All values are reported in ‰. | | | | | | | | | | | |
| --- | --- | --- | --- | --- | --- | --- | --- | --- | --- | --- | --- |
|  | *δ*^15^N^α^ |  | *δ*^15^N^β^ |  | *δ*^15^N^SP^ |  | *δ*^15^N |  | *δ*^18^O |  | *n* |
| RM1A | +0.15 | 0.41 | +1.19 | 0.81 | −1.04 | 0.91 | +0.67 | 0.45 | +39.37 | 1.24 | 5 |
| RM1B | −0.06 | 0.41 | +1.13 | 0.81 | −1.19 | 0.91 | +0.53 | 0.45 | +38.86 | 1.24 | 5 |
| RM2 | +15.81 | 0.41 | −1.19 | 0.81 | +17.00 | 0.91 | +7.31 | 0.45 | +44.08 | 1.25 | 5 |
| RM3A | +51.34 | 0.44 | +55.47 | 0.82 | −4.13 | 0.93 | +53.41 | 0.47 | +103.21 | 1.30 | 5 |
| RM3B | +16.11 | 0.42 | +16.79 | 0.81 | −0.68 | 0.91 | +16.45 | 0.46 | +55.28 | 1.26 | 5 |
| RM4 | +103.17 | 0.45 | +105.92 | 0.82 | −2.75 | 0.93 | +104.54 | 0.50 | +154.35 | 1.36 | 5 |
| RM5 | +43.86 | 0.41 | +23.67 | 0.81 | +20.20 | 0.91 | +33.76 | 0.46 | +39.50 | 1.29 | 5 |

### Table S4

At MPI-BGC (MPI-I), N_2_O RMs, an in-house N_2_O standard gas (NINO), and a number of quality control standards (IAEA-N2, USGS40, Ali-j3, Caf-J3) were analysed for *δ*^15^N by EA-IRMS. EA-IRMS measurements were related to the primary reference material (IAEA‑N1) and the second scale anchor (USGS32) of the* *Air-N_2_ scale. The accuracy of the applied approach was confirmed by the agreement of *δ*^15^N results for the quality control standards, within the stated uncertainties.

| Table S4. *δ*^15^N of RMs, the in-house N_2_O standard gas (NINO), and a number of quality control standards, analysed by Lab MPI (EA-IRMS, Thermo Delta plus, MPI‑I) versus primary reference materials and second scale anchor of the Air-N_2_ scale (IAEA-N1, USGS32). *n* indicates the number of analyses. Expanded uncertainties are calculated following the law of error propagation. For the quality control, standards target values and references are provided as well. All values are reported in ‰. | | | | | | |
| --- | --- | --- | --- | --- | --- | --- |
|  | *δ*^15^N |  | *n* | *δ*^15^N (target) |  | reference |
| RM1A | +0.44 | 0.16 | 38 |  |  |  |
| RM1B | +0.33 | 0.14 | 14 |  |  |  |
| RM2 | +7.09 | 0.16 | 30 |  |  |  |
| RM3A | +53.25 | 0.15 | 10 |  |  |  |
| RM3B | +16.14 | 0.14 | 10 |  |  |  |
| RM4 | +104.39 | 0.37 | 11 |  |  |  |
| RM5 | +33.52 | 0.21 | 14 |  |  |  |
| NINO | +0.54 | 0.21 | 118 | +0.32 | 0.08 | ^11^ |
| IAEA-N2 | +20.41 | 0.18 | 58 | +20.41 | 0.12 | ^12^ |
| USGS40 | −4.60 | 0.17 | 94 | −4.52 | 0.06 | ^12^ |
| Ali-j3 | −1.54 | 0.20 | 246 | −1.51 | 0.1 |  |
| Caf-J3 | −15.45 | 0.17 | 24 | −15.46 | 0.1 |  |

### Table S5

| Table S5. Isotopic composition of RMs, analysed as N_2_O diluted to 0.09 mmol mol^–1^ on Sercon GEO 20-20 IRMS (UEA-I) after gold decomposition, scale-normalised to the *δ*^18^O value of RM4. *n* indicates the number of analyses. Uncertainties are calculated using the law of error propagation from the standard deviations of replicate measurements against the working reference gas and the calibration uncertainties of the working reference gas against Air-N_2_ and VSMOW.^14^ All values are reported in ‰. | | | | | | | | | |
| --- | --- | --- | --- | --- | --- | --- | --- | --- | --- |
|  | *δ*^15^N |  | *δ*^18^O |  | *δ*^17^O |  | *Δ*^17^O |  | *n* |
| RM1A | +0.29 | 0.13 | +39.06 | 0.25 | +20.33 | 0.59 | −0.51 | 0.14 | 17 |
| RM1B | +0.24 | 0.10 | +38.77 | 0.24 | +20.88 | 0.56 | −0.47 | 0.34 | 16 |
| RM2 | +6.73 | 0.07 | +43.69 | 0.24 | +20.87 | 0.40 | −2.38 | 0.14 | 15 |
| RM3A | +52.69 | 0.11 | +103.04 | 0.27 | +23.78 | 0.54 | −27.75 | 0.45 | 16 |
| RM3B | +15.96 | 0.17 | +54.98 | 0.26 | +21.22 | 0.24 | −7.43 | 0.28 | 19 |
| RM4 | +104.18 | 0.13 | +155.17 | 0.39 | +26.71 | 0.83 | −48.58 | 0.76 | 16 |
| RM5 | +33.38 | 0.10 | +39.43 | 0.24 | +20.90 | 0.44 | −0.26 | 0.19 | 15 |

### Table S6

| Table S6. Isotopic composition of RMs, analysed as pure N_2_O on Finnigan MAT 253 IRMS (UEA-II) using the actual *Δ*^17^O measurements. *n* indicates the number of analyses. Uncertainties are calculated using the law of error propagation from the standard deviations of replicate measurements against the working reference gas and the calibration uncertainties of the working reference gas against Air-N_2_ and VSMOW.^15^ All values are reported in ‰. | | | | | |
| --- | --- | --- | --- | --- | --- |
|  | *δ*^15^N |  | *δ*^18^O |  | *n* |
| RM1A | +0.28 | 0.06 | +39.22 | 0.22 | 5 |
| RM1B | +0.19 | 0.06 | +38.83 | 0.22 | 5 |
| RM2 | +6.94 | 0.06 | +44.02 | 0.22 | 5 |
| RM3A | +53.09 | 0.07 | +102.78 | 0.24 | 5 |
| RM3B | +16.08 | 0.06 | +55.13 | 0.22 | 5 |
| RM4 | +104.30 | 0.08 | +153.63 | 0.24 | 5 |
| RM5 | +33.45 | 0.06 | +39.50 | 0.22 | 5 |

## References

1. Liu D, Fang Y, Tu Y, Pan Y. Chemical method for nitrogen isotopic analysis of ammonium at natural abundance. *Anal Chem.* 2014;86(8):3787-3792.

2. Casciotti KL, Sigman DM, Hastings MG, Böhlke JK, Hilkert A. Measurement of the oxygen isotopic composition of nitrate in seawater and freshwater using the denitrifier method. *Anal Chem.* 2002;74(19):4905-4912.

3. Sigman DM, Casciotti KL, Andreani M, Barford C, Galanter M, Böhlke JK. A bacterial method for the nitrogen isotopic analysis of nitrate in seawater and freshwater. *Anal Chem.* 2001;73(17):4145-4153.

4. Gentile N, Rossi MJ, Delémont O, Siegwolf RTW. *δ*^15^N measurement of organic and inorganic substances by EA-IRMS: A speciation-dependent procedure. *Anal Bioanal Chem.* 2013;405(1):159-176.

5. Felix DJ, Elliott EM, Gish TJ, McConnell LL, Shaw SL. Characterizing the isotopic composition of atmospheric ammonia emission sources using passive samplers and a combined oxidation-bacterial denitrifier approach. *Rapid Commun Mass Spectrom.* 2013;27(20):2239-2246.

6. Lachouani P, Frank AH, Wanek W. A suite of sensitive chemical methods to determine the δ^15^N of ammonium, nitrate and total dissolved N in soil extracts. *Rapid Commun Mass Spectrom.* 2010;24(24):3615-3623.

7. Knowles R, Blackburn H. *Nitrogen Isotope Techniques.* Cambridge, USA: Academic Press.; 1993.

8. Amberger A, Schmidt HL. Natürliche Isotopengehalte von Nitrat als Indikatoren für dessen Herkunft. *Geochim Cosmochim Acta.* 1987;51(10):2699-2705.

9. Böttcher J, Strebel O, Voerkelius S, Schmidt HL. Using isotope fractionation of nitrate-nitrogen and nitrate-oxygen for evaluation of microbial denitrification in a sandy aquifer. *J Hydrol.* 1990;114(3-4):413-424.

10. QMA504-2/7. *Bestimmung des Stickstoff-Isotopenverhältnisses (^15^N/^14^N) an im Wasser gelösten Nitrat und Ammonium mittels Isotopenverhältnis-Massenspektrometrie (IRMS) (Qualitätsmanagement-Arbeitsanweisung).*

11. Ostrom NE, Gandhi H, Coplen TB, et al. Preliminary assessment of stable nitrogen and oxygen isotopic composition of USGS51 and USGS52 nitrous oxide reference gases and perspectives on calibration needs. *Rapid Commun Mass Spectrom.* 2018;32(15):1207-1214.

12. Brand WA, Coplen TB, Vogl J, Rosner M, Prohaska T. Assessment of international reference materials for isotope-ratio analysis (IUPAC technical report). *Pure Appl Chem.* 2014;86(3):425-467.

13. Kaiser J, Röckmann T. Correction of mass spectrometric isotope ratio measurements for isobaric isotopologues of O_2_, CO, CO_2_, N_2_O and SO_2_. *Rapid Commun Mass Spectrom.* 2008;22(24):3997-4008.

14. Kaiser J, Röckmann T, Brenninkmeijer CAM. Complete and accurate mass spectrometric isotope analysis of tropospheric nitrous oxide. *J Geophys Res.* 2003;108(15).

15. Mohn J, Werner RA, Buchmann B, Emmenegger L. High-precision *d*^13^CO_2_ analysis by FTIR spectroscopy using a novel calibration strategy. *J Mol Struct.* 2007;834-836(SPEC. ISS.):95-101.
